# Parallel Cooperative Coevolutionary Grey Wolf Optimizer for Path Planning Problem of Unmanned Aerial Vehicles

**DOI:** 10.3390/s22051826

**Published:** 2022-02-25

**Authors:** Raja Jarray, Mujahed Al-Dhaifallah, Hegazy Rezk, Soufiene Bouallègue

**Affiliations:** 1 Research Laboratory in Automatic Control (LARA), National Engineering School of Tunis (ENIT), University of Tunis El Manar, Tunis 1002, Tunisia; raja.jarray@enit.utm.tn (R.J.); soufiene.bouallegue@issig.rnu.tn (S.B.); 2 Control and Instrumentation Engineering Department, King Fahd University of Petroleum & Minerals, Dhahran 31261, Saudi Arabia; mujahed@kfupm.edu.sa; 3 Interdisciplinary Research Center (lRC) for Renewable Energy and Power Systems, King Fahd University of Petroleum & Minerals, Dhahran 31261, Saudi Arabia; 4 College of Engineering at Wadi Addawaser, Prince Sattam Bin Abdulaziz University, Al-Kharj 11911, Saudi Arabia; 5 High Institute of Industrial Systems of Gabes (ISSIG), University of Gabes, Gabes 6011, Tunisia

**Keywords:** unmanned aerial vehicles, paths planning, large-scale global optimization, grey wolf optimizer, cooperative coevolutionary algorithms, parallel master-slave model, Friedman statistical analyses

## Abstract

The path planning of Unmanned Aerial Vehicles (UAVs) is a complex and hard task that can be formulated as a Large-Scale Global Optimization (LSGO) problem. A higher partition of the flight environment leads to an increase in route’s accuracy but at the expense of greater planning complexity. In this paper, a new Parallel Cooperative Coevolutionary Grey Wolf Optimizer (PCCGWO) is proposed to solve such a planning problem. The proposed PCCGWO metaheuristic applies cooperative coevolutionary concepts to ensure an efficient partition of the original search space into multiple sub-spaces with reduced dimensions. The decomposition of the decision variables vector into several sub-components is achieved and multi-swarms are created from the initial population. Each sub-swarm is then assigned to optimize a part of the LSGO problem. To form the complete solution, the representatives from each sub-swarm are combined. To reduce the computation time, an efficient parallel master-slave model is introduced in the proposed parameters-free PCCGWO. The master will be responsible for decomposing the original problem and constructing the context vector which contains the complete solution. Each slave is designed to evolve a sub-component and will send the best individual as its representative to the master after each evolutionary cycle. Demonstrative results show the effectiveness and superiority of the proposed PCCGWO-based planning technique in terms of several metrics of performance and nonparametric statistical analyses. These results show that the increase in the number of slaves leads to a more efficient result as well as a further improved computational time.

## 1. Introduction

Unmanned Aerial Vehicles (UAVs) have recently become an interesting research topic, due to their strong survivability in many activities such as agricultural, commercial, military, and civilian [1,2,3,4]. To achieve repetitive and hard missions in dangerous environments, path planning is a key task in the UAVs’ control system [5,6,7,8]. The purpose of drones’ path planning is not only to find a collision-free path to reach the target but also to select an optimal flyable path that minimizes some critical goals.

The complexity and hardness of UAVs’ path planning problems are increased due to the increase in optimization factors such as UAV restrictions and environmental restrictions. To deal with this complexity, researchers have gradually moved from using the conventional to non-conventional planning approaches considered more effective. In study [9], the authors proposed an improved Particle Swarm Optimization (PSO) algorithm, by introducing the competition strategy formalism, to solve the 3D path planning for fixed-wing UAVs. In study [10], Jamshidi et al. described a CAN bus-based implementation of an asynchronous distributed multi-master parallel Genetic Algorithm (GA) and PSO metaheuristics to improve the performance and computational time of the UAV path planning task. A path planning approach based on the Water Cycle Algorithm (WCA) to find the optimal or near-optimal path avoiding all obstacles in 2D environments is proposed in [11]. The authors in [12] proposed a comprehensively improved particle swarm optimization to enhance the optimality and rapidity of automatic path planners for autonomous UAV formation systems. In studies [13,14], the authors proposed a new methodology to discover the UAV optimal path planning based on a Multiobjective Multi-Verse Algorithm (MOMVA). The authors in [15] proposed a novel approach to solve the UAV path planning based on a Grey Wolf Optimizer (GWO) by proper choice of optimization models such as the objective function for targets and constraints for obstacles’ avoidance. In study [16], another GWO-based method is proposed to solve the UAV path planning problem formulated as a hard optimization problem under operational constraints in terms of path shortness and smoothness as well as avoidance of obstacles. In the same work, the performance of the proposed parameters-free GWO algorithm is compared to other homologous metaheuristics such as the Crow Search Algorithm (CSA), Differential Evolution (DE), Salp Swarm Algorithm (SSA), and others. In study [17], the researchers proposed an improved Adaptive Grey Wolf Optimization (AGWO) algorithm to solve the 3D path planning of UAVs in a complex environment of material delivery in earthquake-stricken areas. Such an algorithm runs with an adaptive convergence factor and updated positions of the search agents. In study [18], a multi-population Chaotic Grey Wolf Optimizer (CGWO)-based method is investigated to solve the 3D UAVs’ cooperative path planning problem. A chaotic search strategy is introduced in this algorithm to improve the exploration/exploitation capabilities of the search behavior. In study [19], Kumar et al. described a modified version of the conventional GWO algorithm (MGWO) to design and optimize suitable paths for autonomous robots.

Such an above study was carried out to arouse the interest in the GWO algorithm widely applied in the field of UAVs’ path planning. The advantages in terms of simplicity of software implementation, reduced number of the algorithm’s control parameters, and convergence fastness make the GWO one of the most extensively used algorithms in the past three years [20,21,22,23]. The increased number of scientific publications on this topic explains the effectiveness of such a stochastic and parameters-free algorithm for solving various optimization problems. However, it should be pointed out that the GWO algorithm is often unable to escape trapping in local minima and presents a premature convergence, especially for the Large-Scale Global Optimization (LSGO) problems. Like most metaheuristics algorithms, the GWO suffers from the “dimensionality curse” and often fails to solve these hard optimization problems [17]. Thus, a practical implementation of such a metaheuristic algorithm presents a challenge in real-world applications due to its prohibitive computational time and weakness concerning an increased number of decision variables of optimization. Although the cited works [15,16,17,18,19] have been developed to solve the UAVs’ path planning problem based on a GWO algorithm, most of them formulated the planning problem with a small number of decision variables. Since the real-world planning tasks are considered LSGO problems, the quantity of computation increases strongly with the increase of the search space dimension, which implies a high probability of converging towards the local optimum [24]. These limits present a serious challenge for the real-time implementation of such an algorithm to design flyable and collision-free UAV paths. 

To overcome these difficulties, the cooperative co-evolutionary concept of optimization seems an interesting idea to further improve the use of GWO algorithms for LSGO problems, particularly in UAVs’ path planning formalism. Such a design approach presents an effective tools’ panoply for solving hard problems thanks to its decomposition of optimization problems into smaller-dimension sub-components. It is a “divide and conquer” strategy initially proposed by Potter and De Jong in [25]. In the literature, the cooperative coevolutionary approach has been successfully applied for various optimization algorithms such as GA [26], PSO [27], DE [28], Simulated Annealing (SA) [29], Ant Colony Optimization (ACO) [30,31], Firefly algorithm [32], and many others. On the other hand, a large quantity of evaluation, due to the large number of problem decision variables, also implies an increased prohibitive computation time. However, online implementation of the standard GWO algorithm for a real-time path planning problem can be failed or at least become ineffective to achieve the desired performance of planning. To overcome this computation constraint, the parallelization concept can be introduced to reduce the complexity of the planning tasks and further increase the computational time of the investigated GWO algorithm.

Over the past decades, there has been a growing interest in the parallelization of metaheuristics algorithms [33,34,35,36,37,38,39,40]. Such advanced mechanisms for computation accelerating and enhancement greatly contribute to the success of metaheuristics for solving hard and large-scale optimization problems. In the literature, many types of metaheuristics algorithms have been recently parallelized based on different architectures and hardware resources. The Graphics Processing Units (GPUs) and multi-core Central Processing Units (CPUs)-based techniques are the most extensively proposed approaches. In study [33], a model of a vector parallel’s Ant Colony Optimization (ACO) algorithm is proposed using a multi-core SIMD CPU architecture. Each ant is mapped with a CPU core and the tour construction is accelerated by vector instructions. In study [34], a parallel heterogeneous ensemble feature selection method based on the three genetic (GA), particle swarm (PSO), and grey wolf (GWO) metaheuristics is proposed to enhance the performance of machine learning formalism. The hardware implementation is achieved on a multi-core CPU with GPU. In study [35], a parallel GA algorithm on GPU is investigated and compared to a sequential execution on CPU for wireless sensor data acquisition using a team of unmanned aerial vehicles. In study [36], an island model-based parallel GA is proposed and implemented on a GPU for solving the unequal area facility layout problem. In study [37], a comprehensive survey on parallel PSO algorithms is presented along with their strategies and applications. Several platforms and models, mainly the CPU- and GPU-based parallelization strategies, have been described and discussed. Another comprehensive survey on the parallel implementation of metaheuristics but within a multi-objective evolutionary framework is presented in [38]. An up-to-date review of methods and key contributions to such a research field are described. Other various techniques and strategies of metaheuristics parallelization are described and discussed in [39,40].

Based on these observations, the idea of using the parameters-free GWO algorithm, improved with the two concepts of cooperative coevolutionary and parallel computing, remains a promising and encouraging solution to solve the UAVs’ path planning problems. Indeed, in real-world UAVs’ planning applications, the most suited planners are those with fewer tuning of the effective parameters and a high fastness of the computation processing concerning the dynamics of navigation and software/hardware specifications of embedded control units. High performances in terms of computation speediness, shorter and collision-free flyable paths are always requested and recommended. In this work, a new Parallel Cooperative Coevolutionary Grey Wolf Optimizer (PCCGWO) is proposed and successfully applied in solving the UAVs’ path planning problem over large benchmarks and instances of navigation. Such an improved GWO algorithm combines the cooperative coevolutionary and parallelization mechanisms to ensure an efficient partition of the original large-scale search space into multiple sub-spaces with reduced dimensions. The decomposition of the decision variables vector into several sub-components is achieved and multi-swarms are created from the initial population to be later assigned to optimize a part of the path planning procedure formulated as an LSGO problem. The main contributions of this paper are summarized as follows: (1) an intelligent and efficient path planning strategy is elaborated to guide UAV drones to reach the destination position while avoiding a high number of obstacles and threats. (2) A novel parameters-free PCCGWO algorithm based on an efficient parallelization master-slave mechanism is proposed and successfully applied to solve the UAVs’ path planning problem over several flight scenarios. (3) A nonparametric statistical analysis in the sense of Friedman and post hoc tests is carried out to show the effectiveness and superiority of the proposed PCCGWO-based path planning approach.

The remainder of this paper is organized as follows. In Section 2, the path planning problem for unmanned aerial vehicles is formulated as a constrained large-scale optimization problem. Section 3 presents the proposed parallel cooperative coevolutionary grey wolf optimizer as well as its designed master-slave architecture. A pseudo-code of the proposed PCCGWO algorithm is given to solve the formulated UAVs’ path planning problem. In Section 4, demonstrative results over 20 different flight scenarios are carried out and discussed to assess the effectiveness of the proposed planning approach. Section 5 concludes this paper.

## 2. Path Planning Problem Formulation

The planning of a flyable and feasible path is a key task in the formalism of drones’ control and navigation. The general definition of such a problem is the generation of a path that guides the drone from a starting point $A:\left(x_{1},y_{1},z_{1}\right)$ to a predefined destination $B:\left(x_{n},y_{n},z_{n}\right)$. To ensure this, an environmental modeling is required [13,14,16,41]. The *x*-axis range $\left(x_{1},x_{n}\right)$ is divided into n−1 equal segments delimited by geometric perpendicular hyper-planes passing through the corresponding points $\left\{x_{1},x_{2},\ldots ,x_{n}\right\}$. A waypoint $w_{i}=\left(x_{i},y_{i},z_{i}\right)$ will be taken at each perpendicular plane and a sequence of these generated points $\Omega_{AB}=\left\{A,\left(x_{2},y_{2},z_{2}\right),\ldots ,\left(x_{n-1},y_{n-1},z_{n-1}\right),B\right\}$ can be formed. The connection of the different waypoints forming such a flight sequence leads to generating the complete flyable path. In this manner, the path planning problem can be reformulated as an optimization problem that consists in determining the optimal flight waypoints’ sequences minimizing a previously defined performance criterion, i.e., shorter, collision-free and smoother flyable paths [14,41]. In this formulation, the decision variables of such a constrained optimization problem are defined as the vector of coordinates of the waypoints $X=\left(y_{2},y_{3},\ldots ,y_{n-1},z_{2},z_{3},\ldots ,z_{n-1}\right)\in {\mathbb{R}}^{2n-4}$.

For the drones’ navigation, the length of the flyable path is an essential objective. The shorter path can reduce the flight time and extend the battery life which ensures more safety. Therefore, a shorter path remains desirable in all planning problems. To well formulate such a design goal, the corresponding objective function to be minimized is chosen as follows [14,41]:

(1) $$f\left(X\right)=\frac{\sum_{k=1}^{n-1}\sqrt{{\left(x_{k+1}-x_{k}\right)}^{2}+{\left(y_{k+1}-y_{k}\right)}^{2}+{\left(z_{k+1}-z_{k}\right)}^{2}}}{\sqrt{{\left(x_{n}-x_{1}\right)}^{2}+{\left(y_{n}-y_{1}\right)}^{2}+{\left(z_{n}-z_{1}\right)}^{2}}}$$

For any path planning problem, the obstacles’ collision avoidance constraint is a key task. Indeed, to ensure that the planned path is safe, the UAV drone must avoid all obstacles. On the other hand, to avoid the risk of being detected by the radars or missiles, a UAV cannot pass through the dangerous regions and/or fly over them [13,14,16,41]. Thus, such an obstacles’ avoidance constraint is modeled by the following expression:

(2) $$g_{1}\left(X\right)=\left(r_{obs}^{i}+\Delta \right)-\sqrt{{\left(x_{uav}-x_{obs}^{i}\right)}^{2}+{\left(y_{uav}-y_{obs}^{i}\right)}^{2}}\leq 0$$

where $r_{obs}^{i}$ and $\left(x_{obs}^{i},y_{obs}^{i}\right)$ are the radius and position of the *i*th obstacle, respectively; $\left(x_{uav},y_{uav},z_{uav}\right)$ means the coordinate of the UAV drone, and Δ presents the predefined safety distance between the drone and a detected obstacle.

When a UAV performs angle management, it can influence its dynamic characteristics and make its flight operation inefficient. Therefore, the angle between two adjacent segments is introduced to limit the straightness of the path. This performance constraint can be formulated as follows [42]:

(3) $$g_{2}\left(X\right)=\left|\varphi_{q,q+1}\right|-\varphi_{\max}\leq 0$$

where $\varphi_{q,q+1}$ is the angle between the two adjacent *q*th and (*q*+1)th segments connecting the waypoints, and $\varphi_{\max}$ is the maximum value of the steering angle.

Finally, the formulated constrained optimization problem for the UAVs’ path planning procedure is defined as follows:

(4) $$\left\{ \begin{array}{l} \underset{X\in D\subseteq {\mathbb{R}}^{d}}{\mathrm{Minimize}}f\left(X\right)\\ \mathrm{subject}\;\mathrm{to}:\\ g_{1}\left(X\right)\leq 0\\ g_{2}\left(X\right)\leq 0 \end{array}\right.$$

where f(.) is the cost function of Equation (1), $g_{1}\left(.\right)$ and $g_{2}\left(.\right)$ are the constraints given by Equations (2) and (3), respectively, $X\in {\mathbb{R}}^{2n-4}$ is the vector of decision variables, and $D=\left\{X\in {\mathbb{R}}^{d}|X^{\min}\leq X\leq X^{\max}\right\}$ is the bounded d-dimensional search space.

To handle the operational constraints (2) and (3) of the optimization problem (4), a static penalty function-based technique is used as follows [41]:

(5) $$\Phi \left(X\right)=f\left(X\right)+\sum_{i=1}^{n_{con}}\kappa_{i}\max{\left\{0,g_{i}\left(X\right)\right\}}^{2}$$

where $\kappa_{i}$ are the scaling penalty coefficients and $n_{con}$ means the number of constraints. 

## 3. Proposed Parallel Cooperative Coevolutionary Algorithm

### 3.1. Grey Wolf Metaheuristic

The Grey Wolf Optimizer (GWO) is a swarm intelligence-based algorithm that is inspired by the leadership hierarchy and hunting strategy of grey wolves in nature [43]. Three leader wolves named *α*, *β*, and *δ* are considered in the hierarchy of the GWO formalism. The most dominating member among the group is called alpha (*α*), followed by beta (*β*) and delta (*δ*) ones which help to lead the rest of the wolves, considered as omega (*ω*) members, toward promising areas. The *i*th wolf is characterized by its position $x_{k}^{i}=\left(x_{k,1}^{i},x_{k,2}^{i},\ldots ,x_{k,d}^{i}\right)$ in the d-dimensional search space. The prey’s position is denoted as $x_{k}^{p}=\left(x_{k,1}^{p},x_{k,2}^{p},\ldots ,x_{k,d}^{p}\right)$. The best candidate solutions *α*, *β*, and *δ* are characterized by their positions $x_{k}^{best,1}$, $x_{k}^{best,2}$, and $x_{k}^{best,3}$. 

For hunting a prey, the grey wolves follow the following three main steps, i.e., encircling, hunting, and attacking [43]:

− Encircling: To mathematically model the strategy of encircling prey by wolves, the following equations have been proposed:
  (6) $$x_{k+1}^{i}=x_{k}^{p}-\Delta_{k}\vartheta_{k}$$
  (7) $$\Delta_{k}=\left|\lambda_{k}x_{k}^{p}-x_{k}^{i}\right|$$
  (8) $$\vartheta_{k}=2\upsilon_{k}U\left(0,1\right)-\upsilon_{k}$$
  where $\lambda_{k}$ are random numbers between 2 and 0, $\upsilon_{k}$ are linearly decreased from 2 to 0 over the iterations course, and U(0,1) is a uniformly random number in [0, 1].
− Hunting: The best candidate solutions *α*, *β*, and *δ* guide the other* ω* wolves to find the global solution of the prey by updating their positions as follows:
  (9) $$x_{k+1}^{i}=\frac{x_{k}^{best,1}+x_{k}^{best,2}+x_{k}^{best,3}}{3},\;i\neq \alpha ,\beta ,\delta$$
  where $x_{k}^{best,1}=x_{k}^{\alpha }-\Delta_{k}^{\alpha }\vartheta_{1,k}$, $x_{k}^{best,2}=x_{k}^{\beta }-\Delta_{k}^{\beta }\vartheta_{2,k}$, and $x_{k}^{best,3}=x_{k}^{\delta }-\Delta_{k}^{\delta }\vartheta_{3,k}$.
  In Equation (9), the coefficient vectors $\vartheta_{1,k}$, $\vartheta_{2,k}$, and $\vartheta_{3,k}$ as well as $\Delta_{k}^{\alpha }$, $\Delta_{k}^{\beta }$, and $\Delta_{k}^{\delta }$ are computed as follows:
  (10) $$\left\{ \begin{array}{l} \vartheta_{1,k}=2\upsilon_{1,k}U\left(0,1\right)-\upsilon_{1,k};\vartheta_{2,k}=2\upsilon_{2,k}U\left(0,1\right)-\upsilon_{2,k};\vartheta_{3,k}=2\upsilon_{3,k}U\left(0,1\right)-\upsilon_{3,k}\\ \Delta_{k}^{\alpha }=\left|\lambda_{1,k}x_{k}^{\alpha }-x_{k}^{i}\right|;\Delta_{k}^{\beta }=\left|\lambda_{2,k}x_{k}^{\beta }-x_{k}^{i}\right|;\Delta_{k}^{\delta }=\left|\lambda_{3,k}x_{k}^{\delta }-x_{k}^{i}\right| \end{array}\right.$$
  where $\upsilon_{j,k}$, j∈{1,2,3}, are linearly decreased from 2 to 0 over the iterations course and $\lambda_{j,k}$ are random numbers distributed uniformly between 2 and 0.
− Attacking: To mathematically model the prey attack approach, the value $\upsilon_{k}$ is linearly decreased from 2 to 0 during iterations and involves the reduction of the fluctuation range $\vartheta_{k}$ which is a random value in the interval $\left[-2\upsilon_{k},2\upsilon_{k}\right]$. When the value $\left|\vartheta_{k}\right|<1$, the next positions of wolves will be between their current positions and the prey one that may force them to attack. After the attack and at the next iteration, this process is repeated until the termination criterion is verified.

Finally, a pseudo-code of the basic GWO algorithm is presented by Algorithm 1 as given in [16,20,43].
**Algorithm 1:** GWO pseudo-code.1.Randomly initialize the grey wolves’ population.2.Initialize $\vartheta_{j,0}$, $\upsilon_{j,0}$, and $\lambda_{j,0}^{i}$.3.Calculate the objective values for each search agent and select $x_{0}^{\alpha }$, $x_{0}^{\beta }$, and $x_{0}^{\delta }$.4.Update the position of the current search agent.5.Update $\vartheta_{j,k}$, $\upsilon_{j,k}$, and $\lambda_{j,k}^{i}$
6.Calculate the objective values of all search agents by applying Equation (10).7.Update the positions $x_{k}^{\alpha }$, $x_{k}^{\beta }$, and $x_{k}^{\delta }$.8.Check the termination criterion and make iterations repeated.

### 3.2. Cooperative Coevolutionary Concept

In cooperative coevolutionary algorithms, the optimization problem to be solved is divided into sub-components in the search space and each of them is solved independently by a species or a sub-swarm which is managed by a processor. In mono-objective optimization formalism, Potter and De Jong were the first to propose such a model [25]. The decision variables are split into sub-components and each sub-swarm seeks to optimize its component by applying a standard evolutionary algorithm. These sub-swarms share information among themselves during evolution. To assess the quality of its optimization, a species builds a complete solution with the representative of all other species and its dedicated decision subcomponent. This is how they cooperate in evolution. The representative of the sub-swarm can be defined by their current best individual or by a random choice. For a given sub-swarm, the solutions consist of a fixed part and a variable part to be optimized. The cooperative coevolutionary approach consists of three main steps [25]:

i. Decomposing the problem: The vector of decision variables is decomposed into smaller sub-components which can be handled by certain evolutionary algorithms.
ii. Optimizing sub-components: Each sub-component will be evolved separately using an evolutionary algorithm until the stopping criteria are met. This means that each sub-component will be optimized by a sub-swarm.
iii. Co-adapting sub-components: Since sub-components can be interdependent, co-adaptation is necessary to take these interdependencies into account. They share information among themselves during the evolution process.

### 3.3. Parallel Master-Slave Model

**Figure 1:**
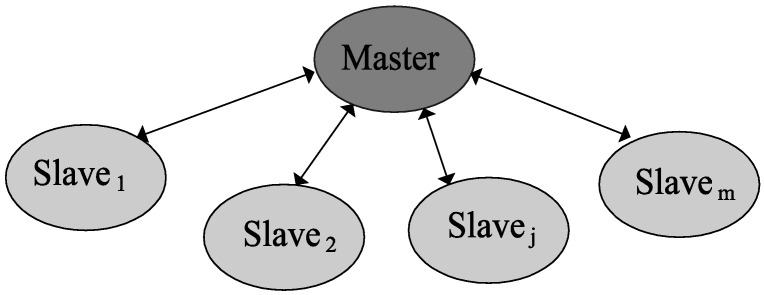
Master-slave model setup.

The master-slave model is one of the most popular approaches for parallel computing due to the simple exploitation of the parallelization capabilities of modern computer systems and its simplicity of implementation. In study [44], Bethke is the first to describe a parallel implementation of an evolutionary algorithm. Subsequently, Grefenstette proposed [45] several prototypes of the parallel evolutionary algorithms representing several variations of the master-slave models. A master-slave model implementation generally requires essential knowledge of the corresponding computer system and minor programming effort. In the master-slave model, one of the processors or cores is selected as the master and the others as slaves of such a master core as shown in Figure 1. The master assigns the slaves hard work or heavy computing tasks and then receives the results from them. The different slaves perform their tasks simultaneously and there is no communication requirement between them. The parallel master-slave model allows a significant reduction in the total computing time required by the algorithm. In such a model of m∈ℕ slaves, the simultaneous evaluation of m individuals is possible, which leads to a significant reduction in the total evaluation time of the population. The parallel software implementation will be more meaningful in large-scale optimization problems [33,34,35,36,37,38,39,40].

### 3.4. Proposed Parallel Cooperative Coevolutionary Grey Wolf Optimizer

The standard GWO algorithm, initially proposed by Mirjalili in 2014, is prone to convergence prematurity. It is also unable to escape local minima in complex multidimensional optimization problems due to its suffering from the “dimensionality curse”. To overcome these challenges, a Cooperative Coevolutionary Grey Wolf Optimizer (CCGWO) is proposed and used to solve the UAVs’ path planning problem formulated as a large-scale optimization one. The original d-dimensional search space is decomposed into m∈ℕ smaller-dimension subspaces $D_{j}$ denoted as follows:

(11) $$D=D_{1}\cup D_{2}\cup \ldots \cup D_{m}$$

Each sub-space should be evaluated by a corresponding sub-swarm. Their dimensions are denoted by $d_{1},d_{2},\ldots ,d_{m}$ which should verify the following condition:

(12) $$d=\sum_{j=1}^{m}d_{j},\;d_{j}\geq 1,\;j=1,2,\ldots ,m$$

where d is the dimension of the original optimization problem. 

The standard GWO algorithm employs a single d-dimensional swarm, but the CCGWO one uses m sub-swarms denoted as $S_{1},S_{2},\ldots ,S_{m}$. Each of them ensures the optimization in the corresponding subspace $D_{j}$ of dimension $d_{j}<d$. The size of a given sub-swarm $S_{j}$ is denoted as $n_{S_{j}}=\left|S_{j}\right|$.

The research agents’ evaluation in each sub-swarm of the CCGWO algorithm is identical to that in the standard GWO one as described in Section 3.1. However, this can pose a significant problem. The agents cannot be updated with the objective function due to the missing components. To overcome this problem, a shared buffer vector, also called a context vector, is defined and contains the complete solution by combining all representatives of sub-swarms [27]. This vector provides the missing information required for each particle or research agent to update with the objective function. Let us consider $c^{\left[j\right]}$ the representative of $d_{j}$-dimensional for sub-swarm $S_{j}$:

(13) $$c^{\left[j\right]}=\left(c_{1}^{\left[j\right]},c_{2}^{\left[j\right]},\ldots ,c_{d_{j}}^{\left[j\right]}\right)$$

The d-dimensional buffer vector C is then obtained by concatenating all different representatives as follows:

(14) $$C=\left(\underset{\mathrm{representative}\;\mathrm{of}\;S_{1}}{\underbrace{c_{1}^{\left[1\right]},\ldots ,c_{d_{1}}^{\left[1\right]}}},\underset{\mathrm{representative}\;\mathrm{of}\;S_{2}}{\underbrace{c_{1}^{\left[2\right]},\ldots ,c_{d_{2}}^{\left[2\right]}}},\ldots ,\underset{\mathrm{representative}\;\mathrm{of}\;S_{m}}{\underbrace{c_{1}^{\left[m\right]},\ldots ,c_{d_{m}}^{\left[m\right]}}}\right)$$

The *i*th research agent of the *j*th sub-swarm of CCGWO, as given by Equation (15), is evaluated by completing the missing components from the buffer vector C:

(15) $$x_{i}^{\left[j\right]}=\left(x_{i,1}^{\left[j\right]},x_{i,2}^{\left[j\right]},\ldots ,x_{i,d_{j}}^{\left[j\right]}\right)\in D_{j}$$

To achieve this, the components $x_{i}^{\left[j\right]}$ are replaced in the buffer’s components that correspond to the representative of the *j*th sub-swarm by keeping the rest unchanged. Hence, the cost value attributed to $x_{i}^{\left[j\right]}$ is defined as:

(16) $$f_{i}^{\left[j\right]}=f\left(C_{i}^{\left[j\right]}\right)$$

where $C_{i}^{\left[j\right]}=\left(\underset{\mathrm{unchanged}}{\underbrace{c_{1}^{\left[1\right]},\ldots ,c_{d_{1}}^{\left[1\right]}}},\ldots ,\underset{\mathrm{considered}\;\mathrm{particle}}{\underbrace{x_{i,1}^{\left[j\right]},\ldots ,x_{i,d_{j}}^{\left[j\right]}}},\ldots ,\underset{\mathrm{unchanged}}{\underbrace{c_{1}^{\left[m\right]},\ldots ,c_{d_{m}}^{\left[m\right]}}}\right)$ with $i=1,2,\ldots ,n_{S_{j}}$.

**Figure 2:**
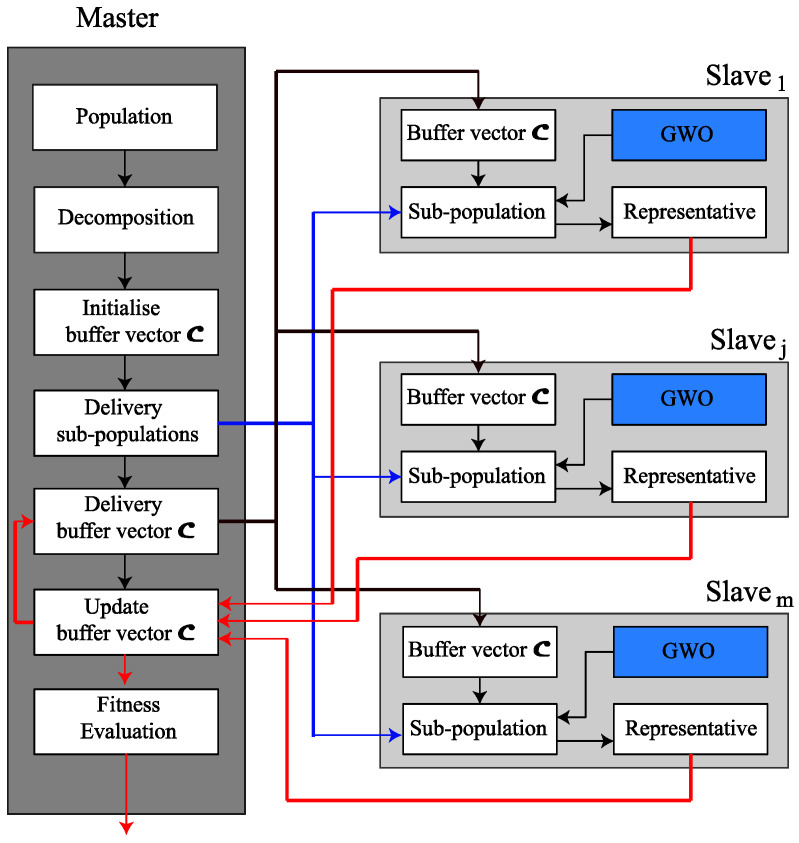
Master-slave modeling of the parallel cooperative coevolutionary grey wolf optimizer.

The representative of each sub-swarm is defined as its best current individual. To parallelize this described cooperative coevolutionary GWO algorithm without changing its co-evolutionary characteristics, a parallel master-slave model is established, resulting in the proposed PCCGWO algorithm as depicted in Figure 2. 

With more details, the master processor will be responsible for initializing the population of research agents, then breaking it down into a set of sub-swarms. Each of them will evolve on part of the problem as a sub-component. The master processor also initializes the buffer vector C using randomly selected individuals from each sub-swarm. After that, it sends to each slave a sub-swarm as well as the buffer vector C. Each slave is designed to evolve a sub-swarm that seeks to optimize its component by applying a standard GWO algorithm for a finite number of times. Such a slave sends the best individuals as representatives to the master after the evolution cycle. The master will build a buffer vector C by concatenating the different representatives and sending it to the different slaves for a new cycle. The master always checks the stop condition, if it is reached, this process will stop. Otherwise, it sends the buffer vector C to all the slaves and asks them to continue the evolutionary process. Finally, Algorithm 2 provides the pseudo-code of the proposed PCCGWO algorithm.
**Algorithm 2:** PCCGWO pseudo-code.Master process1.Randomly initialize the grey wolf population.2.Decompose the population into m sub-swarms denoted as $S_{1},S_{2},\ldots ,S_{m}$.3.Initialize the buffer C using randomly selected individuals from each sub-swarm.4.Send each sub-swarm to a slave.5.Cycle = 06. **While** termination criteria = false **do**7.  **Parallel for** j=1:m slaves8.  Send to slaves the buffer vector C defined in Equation (14).9.  Waiting for slaves.10.  Receive all representatives of sub-swarms from slaves.11.  **End Parallel for**12.  Update the buffer vector C.13.  Cycle = Cycle + 114. **End While**Slave [j] process15. **While** true **do**16.  Receive the buffer vector C from Master process.17.  Execute GWO on sub-swarm $S_{j}$.18.  Send the representative of sub-swarm $S_{j}$ to Master process.19. **End While**

### 3.5. PCCGWO for the UAVs’ Path Planning Problem 

**Figure 3:**
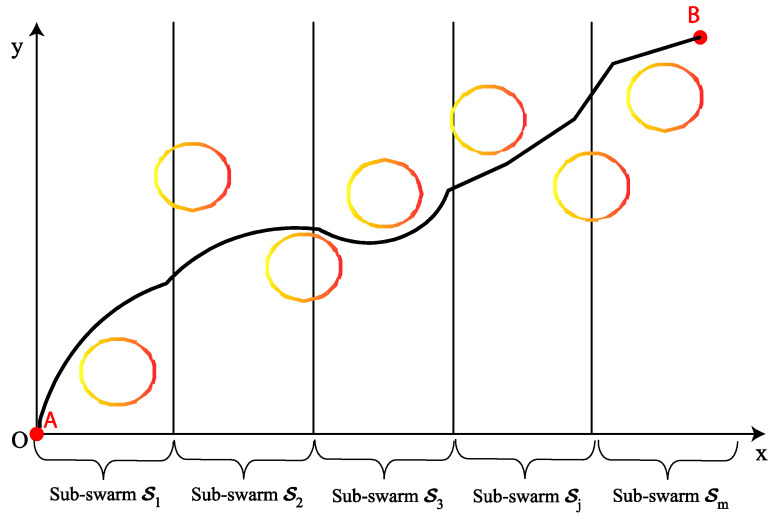
Sketch map of the problem decomposition task.

In the decision variables vector $X=\left(y_{2},y_{3},\ldots ,y_{n-1},z_{2},z_{3},\ldots ,z_{n-1}\right)$, the partition rate n∈ℕ is shown as an important design parameter. Such an effective parameter can significantly affect the performance of the optimization algorithm PCCGWO. The more the n number increases, the dimension of the optimization problem increases, thus leading to an increase in the search complexity. Indeed, a higher partition rate will lead to greater route accuracy and greater planning problem complexity. The original d-dimensional search space is decomposed into equal m smaller-dimension sub-spaces. In this problem, the global path is divided into m sub-paths and each sub-component represents a part of the path. Each sub-population is associated to generate the corresponding sub-path as shown in Figure 3. 

To start optimization with the PCCGWO algorithm, the initial population with the size $n_{pop}$ is generated as follows:

(17) $$Pop_{wolves}=\left[\begin{array}{cccc} y_{1,2} & y_{1,3} & \ldots & y_{1,n-1}\\ y_{2,2} & y_{2,3} & \ldots & y_{2,n-1}\\ \vdots & \vdots & \vdots & \vdots \\ y_{n_{pop},2} & y_{n_{pop},3} & \ldots & y_{n_{pop},n-1} \end{array}\begin{array}{cccc} z_{1,2} & z_{1,3} & \ldots & z_{1,n-1}\\ z_{2,2} & z_{2,3} & \ldots & z_{2,n-1}\\ \vdots & \vdots & \vdots & \vdots \\ z_{n_{pop},2} & z_{n_{pop},3} & \ldots & z_{n_{pop},n-1} \end{array}\right]$$

Such an initial population is decomposed into m sub-swarms $S_{j}$. Each of them is associated to evaluate the corresponding sub-component as follows:

(18) $$\begin{array}{l} S_{1}=\left[\begin{array}{cccc} y_{1,2}^{\left[1\right]} & y_{1,3}^{\left[1\right]} & \ldots & y_{1,(d_{1}/2)+1}^{\left[1\right]}\\ y_{2,2}^{\left[1\right]} & y_{2,3}^{\left[1\right]} & \ldots & y_{2,(d_{1}/2)+1}^{\left[1\right]}\\ \vdots & \vdots & \vdots & \vdots \\ y_{n_{S_{1}},2}^{\left[1\right]} & y_{n_{S_{1}},3}^{\left[1\right]} & \ldots & y_{n_{S_{1}},(d_{1}/2)+1}^{\left[1\right]} \end{array}\begin{array}{cccc} z_{1,2}^{\left[1\right]} & z_{1,3}^{\left[1\right]} & \ldots & z_{1,(d_{1}/2)+1}^{\left[1\right]}\\ z_{2,2}^{\left[1\right]} & z_{2,3}^{\left[1\right]} & \ldots & z_{2,(d_{1}/2)+1}^{\left[1\right]}\\ \vdots & \vdots & \vdots & \vdots \\ z_{n_{S_{1}},2}^{\left[1\right]} & z_{n_{S_{1}},3}^{\left[1\right]} & \ldots & z_{n_{S_{1}},(d_{1}/2)+1}^{\left[1\right]} \end{array}\right]\\ \;\vdots \\ S_{j}=\left[\begin{array}{cccc} y_{1,\left(\sum_{i=1}^{j-1}\left(d_{i}/2\right)\right)+2}^{\left[j\right]} & y_{1,\left(\sum_{i=1}^{j-1}\left(d_{i}/2\right)\right)+3}^{\left[j\right]} & \ldots & y_{1,\left(\sum_{i=1}^{j}\left(d_{i}/2\right)\right)+1}^{\left[j\right]}\\ y_{2,\left(\sum_{i=1}^{j-1}\left(d_{i}/2\right)\right)+2}^{\left[j\right]} & y_{2,\left(\sum_{i=1}^{j-1}\left(d_{i}/2\right)\right)+3}^{\left[j\right]} & \ldots & y_{2,\left(\sum_{i=1}^{j}\left(d_{i}/2\right)\right)+1}^{\left[j\right]}\\ \vdots & \vdots & \vdots & \vdots \\ y_{n_{S_{j}},\left(\sum_{i=1}^{j-1}\left(d_{i}/2\right)\right)+2}^{\left[j\right]} & y_{n_{S_{j}},\left(\sum_{i=1}^{j-1}\left(d_{i}/2\right)\right)+3}^{\left[j\right]} & \ldots & y_{n_{S_{j}},\left(\sum_{i=1}^{j}\left(d_{i}/2\right)\right)+1}^{\left[j\right]} \end{array}\begin{array}{cccc} z_{1,\left(\sum_{i=1}^{j-1}\left(d_{i}/2\right)\right)+2}^{\left[j\right]} & z_{1,\left(\sum_{i=1}^{j-1}\left(d_{i}/2\right)\right)+3}^{\left[j\right]} & \ldots & z_{1,\left(\sum_{i=1}^{j}\left(d_{i}/2\right)\right)+1}^{\left[j\right]}\\ z_{2,\left(\sum_{i=1}^{j-1}\left(d_{i}/2\right)\right)+2}^{\left[j\right]} & z_{2,\left(\sum_{i=1}^{j-1}\left(d_{i}/2\right)\right)+3}^{\left[j\right]} & \ldots & z_{2,\left(\sum_{i=1}^{j}\left(d_{i}/2\right)\right)+1}^{\left[j\right]}\\ \vdots & \vdots & \vdots & \vdots \\ z_{n_{S_{j}},\left(\sum_{i=1}^{j-1}\left(d_{i}/2\right)\right)+2}^{\left[j\right]} & z_{n_{S_{j}},\left(\sum_{i=1}^{j-1}\left(d_{i}/2\right)\right)+3}^{\left[j\right]} & \ldots & z_{n_{S_{j}},\left(\sum_{i=1}^{j}\left(d_{i}/2\right)\right)+1}^{\left[j\right]} \end{array}\right]\\ \;\vdots \\ S_{m}=\left[\begin{array}{cccc} y_{1,\left(\sum_{i=1}^{K-1}\left(d_{i}/2\right)\right)+2}^{\left[m\right]} & y_{1,\left(\sum_{i=1}^{m-1}\left(d_{i}/2\right)\right)+3}^{\left[m\right]} & \ldots & y_{1,n-1}^{\left[m\right]}\\ y_{2,\left(\sum_{i=1}^{K-1}\left(d_{i}/2\right)\right)+2}^{\left[m\right]} & y_{2,\left(\sum_{i=1}^{m-1}\left(d_{i}/2\right)\right)+3}^{\left[m\right]} & \ldots & y_{2,n-1}^{\left[m\right]}\\ \vdots & \vdots & \vdots & \vdots \\ y_{n_{S_{m}},\left(\sum_{i=1}^{m-1}\left(d_{i}/2\right)\right)+2}^{\left[m\right]} & y_{n_{S_{m}},\left(\sum_{i=1}^{m-1}\left(d_{i}/2\right)\right)+3}^{\left[m\right]} & \ldots & y_{n_{S_{m}},n-1}^{\left[m\right]} \end{array}\begin{array}{cccc} z_{1,\left(\sum_{i=1}^{m-1}\left(d_{i}/2\right)\right)+2}^{\left[m\right]} & z_{1,\left(\sum_{i=1}^{m-1}\left(d_{i}/2\right)\right)+3}^{\left[m\right]} & \ldots & z_{1,n-1}^{\left[m\right]}\\ z_{2,\left(\sum_{i=1}^{m-1}\left(d_{i}/2\right)\right)+2}^{\left[m\right]} & z_{2,\left(\sum_{i=1}^{m-1}\left(d_{i}/2\right)\right)+3}^{\left[m\right]} & \ldots & z_{2,n-1}^{\left[m\right]}\\ \vdots & \vdots & \vdots & \vdots \\ z_{n_{S_{m}},\left(\sum_{i=1}^{m-1}\left(d_{i}/2\right)\right)+2}^{\left[m\right]} & z_{n_{S_{m}},\left(\sum_{i=1}^{m-1}\left(d_{i}/2\right)\right)+3}^{\left[m\right]} & \ldots & z_{n_{S_{m}},n-1}^{\left[m\right]} \end{array}\right] \end{array}$$

with $d=\sum_{j=1}^{m}d_{j}$ and $n_{pop}=\sum_{j=1}^{m}n_{S_{j}}$.

## 4. Results and Discussion

### 4.1. Parallel Computing Environment

To pass from a sequential program to a parallel one, the parallelization process is the most efficient attempt. Parallel computing is a powerful way to speed up conception time and the prototyping process. The implementation of a parallel algorithm is highly dependent on the hardware architecture on which the program will be run, but it is also influenced by the software environment. In this work, the MIMD (Multiple Instruction, Multiple Data) systems are used as shared memory architectures commonly known as the multiprocessor. Such hardware/software architecture corresponds to sets of interconnected processors that share the same memory space. Today, most computers have multiple processors, i.e., containing one or more cores, and therefore fall into the family of multiprocessor systems. In a shared memory system, different cores can run in parallel within a process. Threads have access to the common global memory but have their execution stack. The “Parallel Computing Toolbox” software of MATLAB environment allows doing multithreaded programming [46]. In this work, the simplest “Parfor” structure in the MATLAB tool is used to illustrate this functionality. The workers’ number is equal to the iterations number of the parallel loop. MATLAB workers perform iterations independently of each other. They evolve in parallel in the proposed PCCGWO algorithm (one per sub-population). By using Parfor, workers are anonymous, have their execution stack, and share common global memory.

### 4.2. Numerical Experimentations

To illustrate the performance of the proposed PCCGWO algorithm to solve the formulated UAVs’ path planning problem, numerical experimentations with six versions of PCCGWO are carried out and discussed. These proposed PCCGWO versions implement algorithms with different sub-populations equal to 2, 4, 6, 8, 10, and 12. These parallel cooperative coevolutionary algorithms with different sub-swarms are denoted as PCCGWO-2, PCCGWO-4, PCCGWO-6, PCCGWO-8, PCCGWO-10, and PCCGWO-12. In this work, the performances of the proposed PCCGWO algorithms in terms of solution quality and computational speedup are compared to those of the standard GWO one. The effectiveness of the proposed versions of PCCGWO in solving the path planning problem is presented and analyzed under 20 different flight scenarios as described in Table 1.

To assess the effectiveness of the proposed planning approach, these 20 scenarios are different from each other in terms of the number and position of the obstacles as well as the dimension of the planning problem. The problem dimension and obstacles’ number are increased over the scenarios to increase the complexity and hardness of the flight mission. To have equitable and reliable comparisons, the common parameters of the proposed PCCGWO algorithms such as the population size and the maximum number of iterations are set as $n_{pop}=1200$ and $n_{iter}=1500$, respectively. The proposed parallel cooperative coevolutionary algorithms are coded under the MATLAB 2016a environment, and executed on a computer with a Core i5 processor, having 12 cores at 2.90 GHz and 8.00 GB of RAM.

#### 4.2.1. Solution Quality’s Analysis

The proposed parallel cooperative coevolutionary algorithms are performed on the formulated path planning problem given by Equation (4). The six versions of PCCGWO are, however, compared with the standard version of the GWO metaheuristic for the considered different flight scenarios. Three performance criteria such as the value of standardized costs, the path length, and the threats’ avoidance capability are used in each scenario to assess the solution quality. All the proposed GWO and PCCGWO algorithms are executed 20 times independently in each scenario in Table 1. The statistical results of the numerical experimentations under 20 independent runs are summarized in Table 2. All the proposed algorithms are compared based on the objective function value obtained in the best, worst, and mean optimization cases. A smaller standard deviation (STD) value indicates better reproducibility of the optimization algorithm across independent optimization tests. On the other hand, the threats’ avoidance capability of the reported algorithms is quantified by the computation of the PF (Path’s Feasibility) metric. Such a performance index means the percentage of the feasible paths satisfying the operational constraints of the planning problem, i.e., non-collision flight.

While considering the length of the flyable path as an optimization criterion of the problem (4), the investigated Straight-Line Rate (SLR) index is defined as follows:

(19) SLR=path length/|AB|

where |AB| is the straight line’s length between starting point *A* and destination *B*.

A smaller value of the SLR index indicates a better efficiency of the used planning algorithm. The statistical results of numerical experiments over the considered 20 flight instances and under 20 independent runs are summarized in Table 3. All versions of the proposed PCCGWO algorithm are compared to the standard GWO.

From the statistical results of Table 2 and Table 3, one can observe that the best mean cost values and SLR performance indexes are often obtained with the variants of the algorithm with the highest number of slaves, i.e., PCCGWO-10 and PCCGWO-12 ones. Indeed, for this large planning benchmark with 20 instances, as the dimension of the planning problem and the number of obstacles increase, the PF metric decreases for most variants of the PCCGWO algorithms, except those having more increased slaves in their parallel computation mechanisms. Finding a feasible path becomes more difficult when the number of slaves is reduced for instances with high numbers of obstacles and problem dimensions. The proposed PCCGWO-12 algorithm with 12 slaves becomes, on average, the best performing algorithm with tighter forms of the SLR data distribution over the different instances, followed by the PCCGWO-10 and PCCGWO-8 ones.

**Figure 4:**
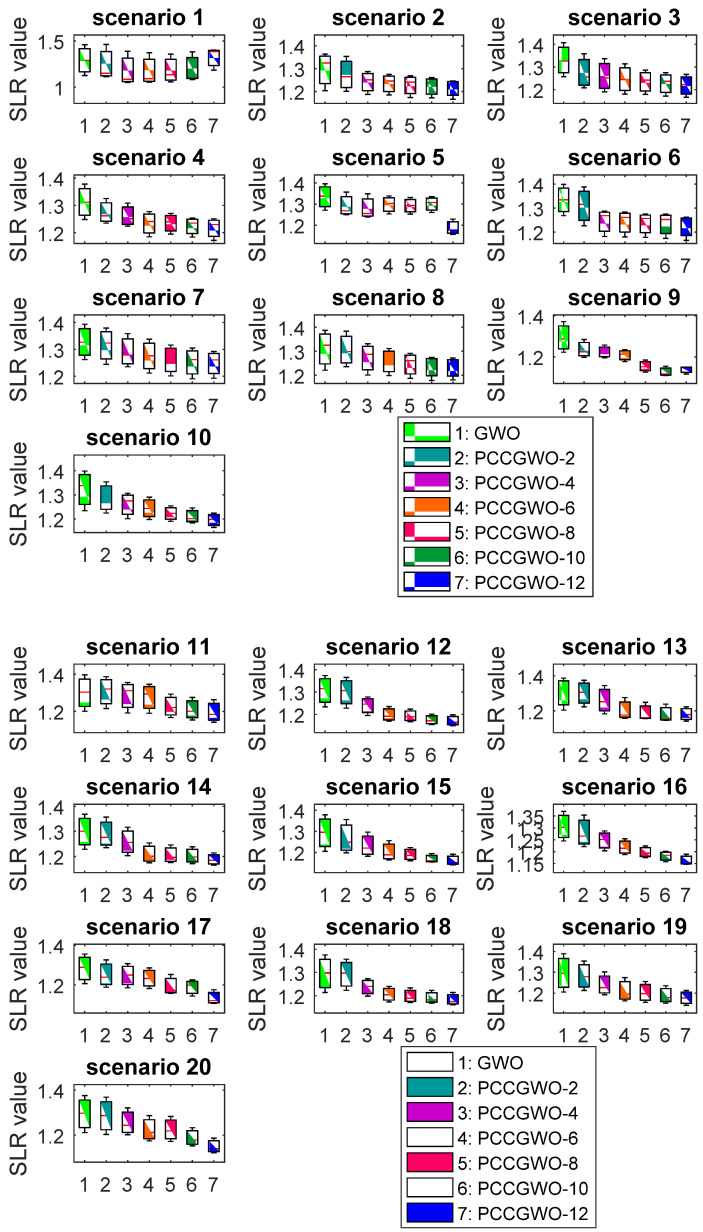
Box-and-Whisker plots of the SLR performance index over the flight scenarios.

On the other hand, Figure 4 shows the Box-and-Whisker plots for the proposed parallel cooperative coevolutionary algorithms over the 20 flight scenarios. In Figure 4, the x-axes of different curves denote the reported algorithms’ names, i.e., 1: GWO, 2: PCCGWO-2, 3: PCCGWO-4, and so on, as shown in the figure’s legend. From these demonstrative results, one can observe that the algorithms with an increased number of slaves, i.e., PCCGWO-10 and PCCGWO-12 variants, often give tighter forms of the SLR data distribution.

**Figure 5:**
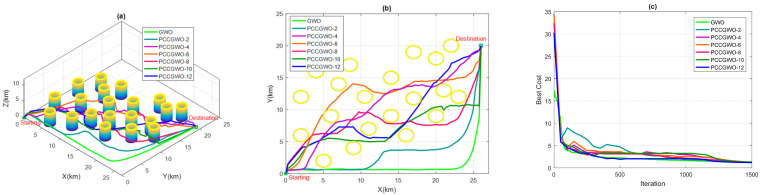
Planning performance in Scenario 5: (**a**) 3D planned paths; (**b**) 2D planned paths; (**c**) Algorithms’ convergence.

**Figure 6:**
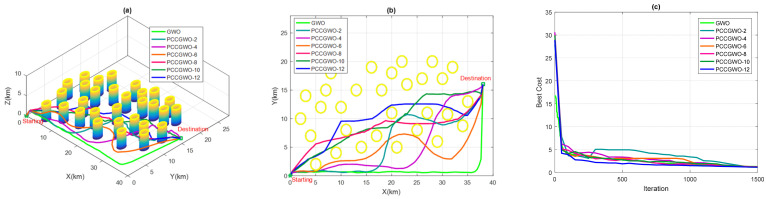
Planning performance in Scenario 9: (**a**) 3D planned paths; (**b**) 2D planned paths; (**c**) Algorithms’ convergence.

**Figure 7:**
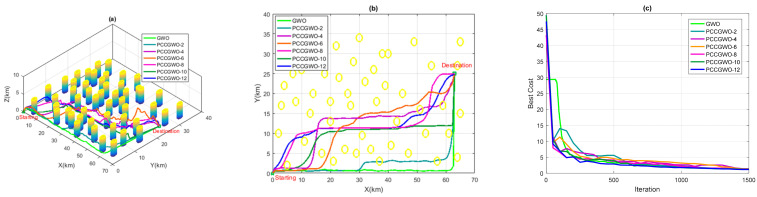
Planning performance in Scenario 17: (**a**) 3D planned paths; (**b**) 2D planned paths; (**c**) Algorithms’ convergence.

**Figure 8:**
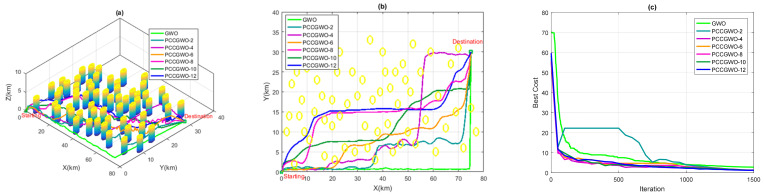
Planning performance in Scenario 20: (**a**) 3D planned paths; (**b**) 2D planned paths; (**c**) Algorithms’ convergence.

On the other hand, and for the threats’ avoidance criterion, some illustrations of the planned paths corresponding to the average case of performance are shown in Figure 5, Figure 6, Figure 7 and Figure 8 for the flight scenarios 5, 9, 17, and 20 of Table 1, respectively. As shown in Figure 5, Figure 6, Figure 7 and Figure 8, all versions of the proposed PCCGWO algorithm are more efficient than the standard GWO in terms of the solution’s quality and fastness convergence. The exploration and exploitation capacities of PCCGWO algorithms are further improved. In scenarios 5 and 9, with problem dimensions equal to 200 and 300, respectively, all versions of the PCCGWO algorithms as well as the standard GWO one give feasible paths and can avoid all obstacles. In scenario 17, with problem dimensions equal to 500, only the PCCGWO-6, PCCGWO-8, PCCGWO-10, and PCCGWO-12 optimizers avoid the danger zones. In scenario 20, with a problem dimension equal to 600 and a high number of obstacles, only the proposed PCCGWO-10 and PCCGWO-12 algorithms give feasible paths. It is obvious that for an increase in the problem dimension, some PCCGWO algorithms become inefficient, due to the fewer number of slaves which become insufficient to provide efficient parallel computing and good research cooperation. In this case, variants of PCCGWO with a higher number of slaves are needed and more sophisticated processors with more than 12 cores are then necessary for these treatments. Additionally, one can observe that the standard GWO never moves between obstacles in the considered flight scenarios. On the contrary, all versions of PCCGWO pass between obstacles to reach the target point. The PCCGWO algorithm remains the more suited solver for performing flight missions with high efficiency compared to the GWO one.

**Figure 9:**
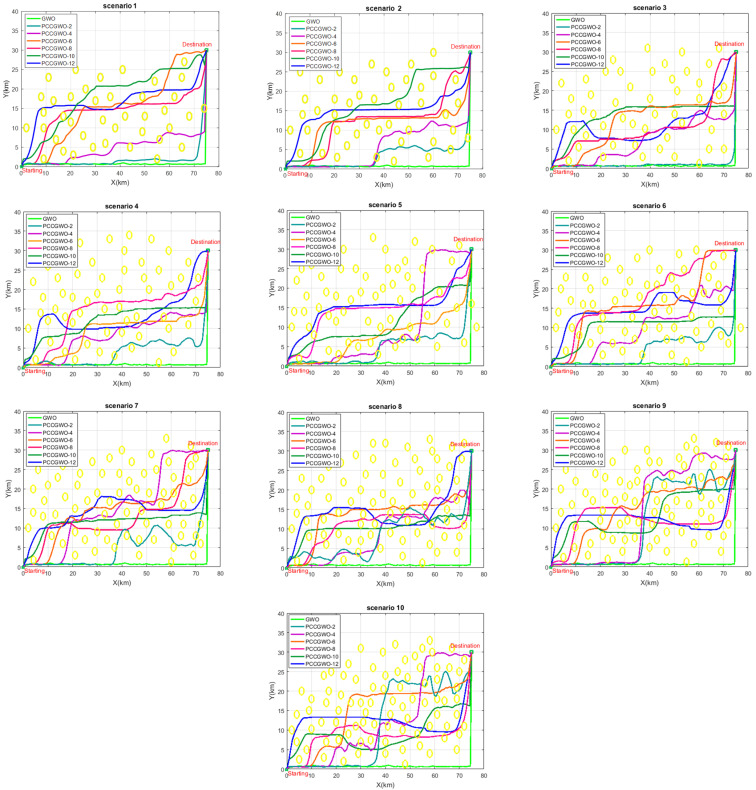
Effect of increasing numbers of PCCGWO’s slaves on the collision-free planning performance.

Let us now analyze the effect of slaves’ number, for a given problem dimension, on the performance of the proposed PCCGWO-based planning process. For this purpose, another 10 flight scenarios, for the same dimension equal to 600 and various numbers and positions of obstacles, are investigated as shown in Table 4. From this result, one can observe that the increase in the number of slaves leads to a decrease in the SLR values. For the threats’ avoidance, the planned paths are shown in Figure 9. In scenarios 1, 2, and 3 of Table 4, with fewer numbers of obstacles, the algorithms PCCGWO-6, PCCGWO-8, PCCGWO-10, and PCCGWO-12 avoid the danger zones. In scenario 4, only the algorithms PCCGWO-8, PCCGWO-10, and PCCGWO-12 give feasible paths. For more complex scenarios, i.e., flight environment with several obstacles, only the PCCGWO-10 and PCCGWO-12 variants give feasible collision-free paths. Thus, for a concrete number of problem dimensions, as the number of obstacles increases, more slaves in the PCCGWO algorithm are needed to find feasible paths. The shorter and collision-free obtained paths confirm the superiority and effectiveness of the proposed PCCGWO optimizers with an increased number of slaves, i.e., PCCGWO-10 and PCCGWO-12 variants. Obviously, with each increase in the dimension of the planning problem, algorithms with more slaves are needed to best handle the complexity of the resulting optimization problem.

Considering the two performance criteria, i.e., standardized cost and SLR, a statistical comparison based on the nonparametric Friedman test is implemented and discussed according to the mean values of performance over 20 different instances. The aim is to statistically study significant differences between the considered PCCGWO variants and standard GWO. For the seven reported algorithms (ζ=7) and the twenty scenarios (η=20), the Iman–Davenport extension of the classical Friedman test [47] leads to the computed value $F_{F_{1}}=52.7465$ for the objective value criterion and $F_{F_{2}}=71.2460$ for the SLR criterion. Based on the F distribution table, the critical value with ζ−1 and (ζ−1)(η−1) degree-of-freedom is equal to $F_{6,114,0.05}=2.1750<F_{F_{1}}<F_{F_{2}}$ at a confidence level of α=0.05. The null hypothesis is therefore rejected and there are significant differences between the performances of the proposed algorithms in solving the path planning problem. Fisher’s LSD post hoc test [48] is applied to find out which algorithms differ from others. The ranks’ sums for all proposed algorithms are summarized in Table 5 and Table 6. When the absolute difference of the ranks’ sum of two algorithms is greater than a critical value, they are declared to be different. Based on the statistical calculation formula given in [48], the critical value is equal to 11.9624 for the standardized cost criterion and 10.6661 for the SLR criterion. Paired comparisons are summarized in Table 7 and Table 8. The underlined values indicate the difference in the performance of the proposed algorithms. From the conducted statistical study, one can see that the standard GWO is the worst performing algorithm according to the standardized cost and SLR criteria of the UAVs’ path planning problem. The six PCCGWO versions surpass the standard GWO in all scenarios with statistical confidence. Indeed, the proposed algorithm PCCGWO-12 becomes the best, followed by PCCGWO-10 and PCCGWO-8 ones. The total number of subpopulations has a big impact on the performance of the PCCGWO algorithms. These demonstrative results show that the proposed PCCGWO algorithm improves the quality of the standard GWO-based solutions.

#### 4.2.2. Computational Time

**Figure 10:**
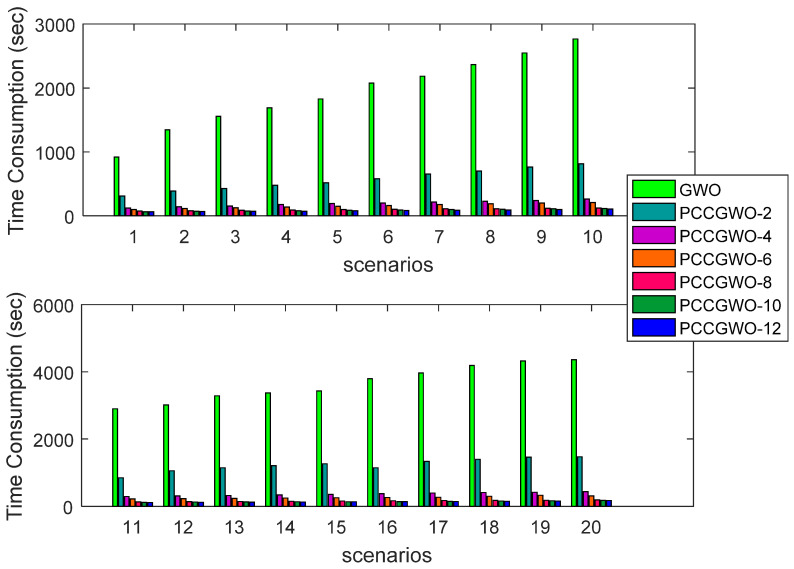
Time consumption performance index’s variations over the 20 flight scenarios.

The performance of the proposed PCCGWO algorithms can be analyzed and compared in terms of the runtime of all reported algorithms over 20 different flight scenarios. The statistical results obtained for the Computational (CT) metric are summarized in Table 9. The obtained runtime measures for the mean case of optimization are also graphically shown in Figure 10. From these demonstrative results, one can notice that the increase in the number of slaves in the parallel master-slave model leads to lower runtimes of the reported PCCGWO algorithms. The PCCGWO-10 and PCCGWO-12 with the highest number of slaves are often the best variants with a remarkable superiority regarding the other reported PCCGWO algorithms. The reason for these fast processing computations is that the population and the decision variables are divided by the number of slaves that are evolved in parallel, i.e., one per subpopulation. It is also noticed that as the size of the problem increases, the runtime increases for all PCCGWO versions. As expected, a heavier computational and communication burden in parallel algorithms may be imposed by the manipulation and transmission of higher dimensional vectors.

#### 4.2.3. Algorithms’ Sensitivity Analysis

In this subsection, a study on the impact of the main control parameters’ settings of the PCCGWO versions, i.e., population size $n_{pop}$ and maximum number of iterations $n_{iter}$, is carried out while considering the path length and the execution time as performance metrics. For this sensitivity analysis of the proposed PCCGWO algorithms, several simulations with different settings of control parameters, as $n_{pop}\in \left\{1200,1600,2000\right\}$, and $n_{iter}\in \left\{1500,2000,2500\right\}$, are performed and summarized in Table 10 and Table 11 for the considered two performance metrics. For a given numerical experimentation, the impact of a single parameter is examined while keeping the other parameter constant. All the performance comparisons are conducted under Scenario 20 of Table 1 which represents the hardest and most complicated path planning instance.

From these demonstrative results, one can notice that the increase in the population size leads to a decrease in the path length and, subsequently, an increase in the execution time for all reported algorithms. It is also obvious that the elapsed time increases linearly with the increase in the number of iterations, on the contrary, the path length decreases. In Scenario 20 of Table 1, the PCCGWO-6, PCCGWO-8, PCCGWO-10, and PCCGWO-12 algorithms give achievable paths while avoiding all the obstacles. With parameters’ setting $n_{pop}=1200$ and $n_{iter}=1500$, only the PCCGWO-10 and PCCGWO-12 algorithms give feasible paths while respecting the collision avoidance constraint. Therefore, as the size population and number of iterations increase, the efficiency of the proposed PCCGWO metaheuristics algorithms improves.

#### 4.2.4. Comparison with Other Metaheuristics Algorithms

To examine and evaluate the performance of the proposed PCCGWO-12, recent and extensively used Water Cycle Algorithm (WCA), Crow Search Algorithm (CSA), Salp Swarm Algorithm (SSA), and Multi-Verse Optimizer (MVO) are considered for the comparison. For these algorithms, the common parameters such as the population size and the maximum number of iterations are set as $n_{pop}=1200$ and $n_{iter}=1500$, respectively. All the performance comparisons are conducted under Scenario 20 of Table 1. All the compared algorithms are independently executed 20 times. The specific control parameters of each reported metaheuristic are summarized as follows:

− WCA [49]: number of rivers: 4, maximum distance: 1 × 10^−16^.
− SSA [50]: no control parameters.
− CSA [51]: awareness probability: 0.2, flight length: 1.
− MVO [52]: min and max of wormhole existence probabilities: 0.2 and 1.

Table 12 presents the optimization results of the compared algorithms in terms of SRL and CT performance criteria. Based on these results, one can observe the superiority of the proposed PCCGWO-12 algorithm in terms of solutions’ quality, results’ reproducibility, and computational speedup, i.e., lower values for the mean SLR criterion, STD indices, and computational time.

**Figure 11:**
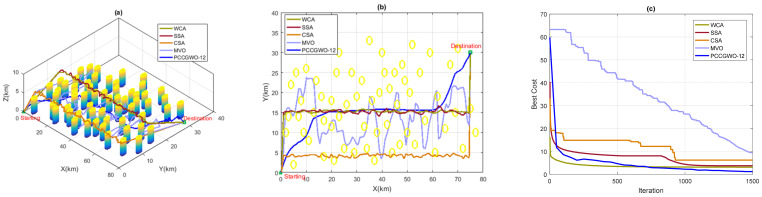
Comparison with WCA, SSA, CSA, and MVO metaheuristics: (**a**) 3D paths; (**b**) 2D paths; (**c**) Algorithms’ convergence.

Figure 11 shows the planned paths of the proposed and compared algorithms. Shorter and collision-free paths are obtained by the PCCGWO-12 planner that also better performs the fastest computation processing. On the contrary, all other reported algorithms are not efficient enough for the considered planning problem with increased numbers of obstacles and dimensions. Some of these planners lead to not flyable paths that traverse the threat zones with a lot of fluctuations. This weakness of WCA, SSA, CSA, and MVO algorithms in the planning process is due to their “dimensionality curse” that often makes failure to solve such large-scale optimization problems. In addition, the exploration and exploitation capacities of the proposed PCCGWO-12 algorithm are superior compared to those of the reported WCA, SSA, CSA, and MVO algorithms. Based on these established comparisons and observations, the superiority and effectiveness of the proposed PCCGWO-based path planning approach are further improved in terms of collision avoidance, shorter planned paths, and fastness of the computation processing. The novelty and originality of our work are well clarified compared to approaches using similar techniques.

## 5. Conclusions

In this paper, a new Parallel Cooperative Coevolutionary variant of the Grey Wolf Optimizer (PCCGWO) based on a parallelization master-slave model has been proposed and successfully applied to solve the UAVs’ path planning problem over large benchmarks and instances of navigation. To overcome the limits and drawbacks of the standard GWO for solving large-scale and complex path planning problems, particularly in terms of dimensionality curse and prohibitive time consuming, two improvement mechanisms in terms of parallelization and cooperative co-evolutionary search are introduced in the proposed PCCGWO algorithm. The UAVs’ path planning problem is formulated as an LSGO problem under operational constraints mainly in terms of obstacles’ collision avoidance and path’s straightness. A cooperative coevolutionary mechanism is applied to make an efficient partition of the original search space into smaller dimensional sub-spaces. The decision variables’ vector is decomposed into several subcomponents with reduced dimensions. An efficient parallelization master-slave technique is then proposed to further reduce the computation time faced with the large-scale and hardness of the planning problem. Six PCCGWO variants with an increased number of slaves, i.e., PCCGWO-2, PCCGWO-4, PCCGWO-6, PCCGWO-8, PCCGWO-10, and PCCGWO-12, are proposed according to the number of the partitioned sub-populations and the available cores of the computer CPU’s processor. Each slave of such a parallel architecture is designed to evolve a sub-swarm that seeks to optimize its component by applying a standard GWO algorithm. The master builds a buffer vector by concatenating the different representatives from slaves, shown as best search agents, and sending it again for a new cycle. The performance analysis of the proposed PCCGWO planners is carried out based on several experiments over different flight instances as well as a comparative study with the standard GWO algorithm, and other recent and extensively used metaheuristics, i.e., Water Cycle Algorithm (WCA), Crow Search Algorithm (CSA), Salp Swarm Algorithm (SSA), and Multi-Verse Optimizer (MVO). The demonstrative results, as well as the nonparametric statistical analyses in the sense of Friedman and post hoc tests, show the effectiveness and superiority of the proposed PCCGWO algorithms with the highest number of slaves, i.e., PCCGWO-10 and PCCGWO-12 variants. The performance metrics in terms of shorter and collision-free planned paths and computational speedup are significantly improved. Obviously, with each increase in the planning problem dimension and number of obstacles, i.e., a more intensive partition of the flight environment, PCCGWO variants with more slaves are needed to best handle the complexity of the resulting optimization problem. As the most suitable drone planners are the ones that have the least parameters’ tuning with an increased computation speediness regarding the software/hardware specifications of the onboard control units, the proposed PCCGWO algorithm can be considered as a promising method for providing shorter and collision-free flight paths in real-world environments.

Future works deal with the implementation of the proposed PCCGWO-based path planning method using the real-world Parrot AR. Drone 2.0 prototype of UAVs and the associated MATLAB/Simulink software. The real-world implementation and prototyping of such a planning algorithm will be investigated regarding all engineering details and managerial implications.

## Figures and Tables

**Table 1:** Information on external installations of the flight environment.

Scenario	Starting Point (km)	Destination Point (km)	Threads’ Number	Dimension
1	(0,0,0)	(13,11,0)	10	100
2	(0,0,0)	(16,13,0)	12	125
3	(0,0,0)	(19,15,0)	15	150
4	(0,0,0)	(22,15,0)	17	175
5	(0,0,0)	(26,20,0)	20	200
6	(0,0,0)	(28,17,0)	22	225
7	(0,0,0)	(32,16,0)	25	250
8	(0,0,0)	(35,17,0)	27	275
9	(0,0,0)	(38,16,0)	30	300
10	(0,0,0)	(41,17,0)	32	325
11	(0,0,0)	(44,20,0)	35	350
12	(0,0,0)	(47,20,0)	37	375
13	(0,0,0)	(50,25,0)	40	400
14	(0,0,0)	(53,25,0)	42	425
15	(0,0,0)	(56,25,0)	45	450
16	(0,0,0)	(60,25,0)	47	475
17	(0,0,0)	(63,25,0)	50	500
18	(0,0,0)	(66,30,0)	52	525
19	(0,0,0)	(69,30,0)	55	550
20	(0,0,0)	(75,30,0)	60	600

**Table 2:** Optimization results of the problem (4): standardized cost criterion.

Scenario		GWO	PCCGWO-2	PCCGWO-4	PCCGWO-6	PCCGWO-8	PCCGWO-10	PCCGWO-12
1	Best	1.1243	1.1198	1.0514	1.1721	1.0725	1.0386	1.1830
	Mean	1.2544	1.1477	1.0836	1.3007	1.1210	1.1337	1.4000
	Worst	1.4501	1.4712	1.3823	1.3761	1.3589	1.3832	1.4652
	STD	0.2822	0.2622	0.1957	0.1937	0.1844	0.1822	0.1813
	PF (%)	55	75	85	85	85	90	90
2	Best	1.2062	1.2132	1.1832	1.1821	1.1739	1.1747	1.1692
	Mean	1.3298	1.2784	1.2587	1.2477	1.2454	1.2498	1.2254
	Worst	1.3680	1.3529	1.2841	1.2783	1.2674	1.2683	1.2446
	STD	0.0887	0.0698	0.0587	0.0492	0.0488	0.0487	0.0393
	PF (%)	55	75	85	85	85	90	90
3	Best	1.1654	1.1262	1.1022	1.0765	1.0865	1.0923	1.0921
	Mean	1.3235	1.2659	1.2514	1.2474	1.2414	1.2345	1.2212
	Worst	1.4087	1.3674	1.3568	1.3356	1.3149	1.3116	1.3066
	STD	0.1246	0.1215	0.1128	0.1127	0.1112	0.1072	0.1042
	PF (%)	50	70	80	80	80	85	85
4	Best	1.2433	1.2387	1.2220	1.1887	1.1936	1.1814	1.1769
	Mean	1.3312	1.2589	1.2498	1.2442	1.2365	1.2341	1.2219
	Worst	1.3714	1.3258	1.3036	1.2774	1.2792	1.2581	1.2526
	STD	0.0678	0.0474	0.0425	0.0448	0.0429	0.0391	0.0385
	PF (%)	50	70	80	80	80	85	85
5	Best	1.2704	1.2341	1.2232	1.2103	1.2154	1.2636	1.1552
	Mean	1.3391	1.2693	1.2608	1.3132	1.2971	1.3253	1.1804
	Worst	1.3910	1.3698	1.3558	1.3641	1.3123	1.3842	1.2236
	STD	0.0658	0.0738	0.0683	0.0674	0.0521	0.0603	0.0626
	PF (%)	45	65	75	75	80	80	80
6	Best	1.2671	1.2236	1.1885	1.1714	1.1746	1.1701	1.1673
	Mean	1.3288	1.3144	1.2656	1.2598	1.2545	1.2487	1.2423
	Worst	1.3918	1.3846	1.2895	1.2787	1.2628	1.2736	1.2614
	STD	0.0678	0.0814	0.0574	0.0571	0.0495	0.0541	0.0487
	PF (%)	45	65	75	75	80	80	80
7	Best	1.2154	1.1765	1.1664	1.1535	1.1515	1.1513	1.1512
	Mean	1.3254	1.3225	1.2787	1.2714	1.2663	1.2643	1.2635
	Worst	1.3987	1.3747	1.3565	1.3565	1.3571	1.3441	1.3412
	STD	0.0969	0.1046	0.1036	0.1031	0.1023	0.1012	0.0953
	PF (%)	45	65	75	75	80	80	80
8	Best	1.2278	1.2341	1.2023	1.1898	1.1814	1.1713	1.1796
	Mean	1.3245	1.3012	1.2844	1.2714	1.2655	1.2532	1.2564
	Worst	1.3812	1.3787	1.3356	1.3082	1.2978	1.2771	1.2732
	STD	0.0795	0.0745	0.0671	0.0614	0.0610	0.0546	0.0485
	PF (%)	45	65	70	70	75	75	75
9	Best	1.2245	1.1845	1.1823	1.1741	1.1321	1.1036	1.0987
	Mean	1.2880	1.2258	1.2168	1.2136	1.1524	1.1171	1.1488
	Worst	1.3547	1.2851	1.2712	1.2548	1.1982	1.1654	1.1632
	STD	0.0654	0.0512	0.0456	0.0401	0.0337	0.0312	0.0341
	PF (%)	40	60	70	70	75	75	75
10	Best	1.2382	1.2241	1.2141	1.1941	1.1974	1.1814	1.1646
	Mean	1.3345	1.3011	1.2784	1.2365	1.2213	1.1988	1.1865
	Worst	1.3952	1.3554	1.3146	1.2936	1.2598	1.2462	1.2263
	STD	0.0787	0.0667	0.0517	0.0497	0.0320	0.0334	0.0311
	PF (%)	35	55	65	70	75	75	75
11	Best	1.1987	1.2122	1.1954	1.1933	1.1642	1.1502	1.1465
	Mean	1.3300	1.3297	1.3222	1.3160	1.2574	1.2149	1.1904
	Worst	1.3954	1.3798	1.3478	1.3424	1.2977	1.2698	1.2564
	STD	0.1002	0.0861	0.0815	0.0792	0.0684	0.0598	0.0552
	PF (%)	35	50	65	65	70	70	70
12	Best	1.2365	1.2245	1.1974	1.1676	1.1614	1.1519	1.1476
	Mean	1.3122	1.2798	1.2512	1.2033	1.1825	1.1745	1.1698
	Worst	1.3785	1.3695	1.2763	1.2326	1.2284	1.2046	1.1962
	STD	0.0714	0.0712	0.0414	0.0327	0.0347	0.0273	0.0241
	PF (%)	35	50	65	65	70	70	70
13	Best	1.2154	1.2245	1.1854	1.1544	1.1563	1.1584	1.1322
	Mean	1.3142	1.3014	1.2868	1.1916	1.2013	1.1719	1.2146
	Worst	1.3874	1.3762	1.3465	1.2789	1.2863	1.2541	1.2465
	STD	0.0961	0.0758	0.0825	0.0637	0.0612	0.0517	0.0591
	PF (%)	30	50	60	60	70	70	70
14	Best	1.2254	1.2398	1.2046	1.1841	1.1765	1.1898	1.1636
	Mean	1.3065	1.2788	1.2641	1.2056	1.1958	1.1965	1.1842
	Worst	1.3684	1.3514	1.3236	1.2695	1.2465	1.2445	1.2198
	STD	0.0784	0.0574	0.0584	0.0498	0.0374	0.0284	0.0281
	PF (%)	25	50	60	60	65	70	70
15	Best	1.2136	1.1988	1.1765	1.1699	1.1632	1.1532	1.1412
	Mean	1.2974	1.2537	1.1841	1.1774	1.1765	1.1825	1.1723
	Worst	1.3721	1.3462	1.2654	1.2465	1.2138	1.2132	1.1945
	STD	0.0891	0.0749	0.0499	0.0423	0.0359	0.0301	0.0265
	PF (%)	25	45	60	60	65	65	65
16	Best	1.2456	1.2226	1.2046	1.1836	1.1786	1.1562	1.1410
	Mean	1.2987	1.2687	1.2465	1.2045	1.1874	1.1745	1.1501
	Worst	1.3684	1.3536	1.2876	1.2593	1.2246	1.2082	1.1935
	STD	0.0687	0.0674	0.0445	0.0392	0.0245	0.0254	0.0234
	PF (%)	20	45	60	60	65	65	65
17	Best	1.2032	1.1849	1.2898	1.2263	1.1465	1.1434	1.1132
	Mean	1.2948	1.2474	1.4029	1.3237	1.2059	1.2384	1.1508
	Worst	1.3865	1.3669	1.4563	1.3412	1.2556	1.2514	1.1874
	STD	0.0937	0.0922	0.0852	0.0648	0.0598	0.0592	0.0370
	PF (%)	20	40	55	55	65	65	65
18	Best	1.2146	1.2325	1.1914	1.1746	1.1774	1.1643	1.1503
	Mean	1.3065	1.2874	1.2475	1.2079	1.1866	1.1801	1.1820
	Worst	1.3841	1.3631	1.2765	1.2663	1.2476	1.2287	1.2032
	STD	0.0687	0.0657	0.0428	0.0411	0.0383	0.0336	0.0254
	PF (%)	15	40	50	55	60	60	60
19	Best	1.2032	1.2033	1.1955	1.1865	1.1632	1.1539	1.1432
	Mean	1.2981	1.2915	1.2163	1.2147	1.2055	1.1918	1.1635
	Worst	1.3789	1.3562	1.3124	1.2893	1.2785	1.2312	1.2136
	STD	0.0882	0.0736	0.0663	0.0536	0.0513	0.0375	0.0357
	PF (%)	15	35	50	55	55	60	60
20	Best	2.1423	1.2865	1.2233	1.2133	1.2566	1.2365	1.1566
	Mean	2.8221	1.3604	1.2990	1.2793	1.3415	1.2884	1.2076
	Worst	2.9987	1.4562	1.3651	1.3741	1.3741	1.3456	1.2533
	STD	0.4589	0.0851	0.0747	0.0878	0.0687	0.0587	0.0414
	PF (%)	10	25	50	55	55	60	60

**Table 3:** Optimization results of the problem (4): SLR path length criterion.

Scenario		GWO	PCCGWO-2	PCCGWO-4	PCCGWO-6	PCCGWO-8	PCCGWO-10	PCCGWO-12
1	Best	1.1269	1.1132	1.0526	1.0548	1.0587	1.0822	1.1855
	Mean	1.2889	1.1511	1.0863	1.0920	1.1336	1.1355	1.3845
	Worst	1.4592	1.4632	1.3882	1.3726	1.3565	1.3811	1.4020
	STD	0.2832	0.2723	0.1952	0.1936	0.1845	0.1814	0.1812
2	Best	1.2041	1.2012	1.1874	1.1845	1.1741	1.1721	1.1654
	Mean	1.3254	1.2654	1.2511	1.2455	1.2412	1.2361	1.2354
	Worst	1.3641	1.3541	1.2874	1.2754	1.2687	1.2614	1.2456
	STD	0.0895	0.0723	0.0517	0.0498	0.0484	0.0465	0.0445
3	Best	1.2576	1.2079	1.1910	1.1803	1.1787	1.1720	1.1673
	Mean	1.3261	1.2564	1.2518	1.2445	1.2418	1.2366	1.2223
	Worst	1.4063	1.3580	1.3357	1.3133	1.2862	1.2747	1.2672
	STD	0.0814	0.0706	0.0628	0.0527	0.0483	0.0455	0.0425
4	Best	1.2487	1.2354	1.2239	1.1854	1.1952	1.1841	1.1721
	Mean	1.3121	1.2624	1.2515	1.2411	1.2401	1.2354	1.2301
	Worst	1.3784	1.3254	1.3087	1.2774	1.2711	1.2544	1.2512
	STD	0.0684	0.0414	0.0488	0.0441	0.0397	0.0354	0.0410
5	Best	1.2712	1.2495	1.2394	1.2533	1.2530	1.2599	1.1569
	Mean	1.3381	1.2682	1.2555	1.3026	1.2934	1.3075	1.1773
	Worst	1.3958	1.3565	1.3477	1.3382	1.3310	1.3251	1.2289
	STD	0.0624	0.0515	0.0494	0.0439	0.0342	0.0479	0.0354
6	Best	1.2687	1.2263	1.1814	1.1798	1.1781	1.1741	1.1654
	Mean	1.3345	1.3154	1.2684	1.2611	1.2566	1.2523	1.2465
	Worst	1.3987	1.3874	1.2874	1.2841	1.2754	1.2759	1.2625
	STD	0.0689	0.0884	0.0541	0.0514	0.0516	0.0541	0.0521
7	Best	1.2623	1.2446	1.2355	1.2122	1.2017	1.1910	1.1933
	Mean	1.3268	1.3239	1.2794	1.2766	1.2674	1.2624	1.2611
	Worst	1.3942	1.3798	1.3587	1.3383	1.3165	1.3052	1.2923
	STD	0.0955	0.0660	0.0636	0.0529	0.0418	0.0401	0.0348
8	Best	1.2214	1.2365	1.2014	1.1987	1.1874	1.1788	1.1812
	Mean	1.3255	1.2988	1.2874	1.2654	1.2612	1.2541	1.2443
	Worst	1.3874	1.3841	1.3314	1.3121	1.2914	1.2744	1.2718
	STD	0.0894	0.0644	0.0614	0.0541	0.0531	0.0504	0.0482
9	Best	1.2236	1.1987	1.1967	1.1774	1.1263	1.1099	1.1169
	Mean	1.2880	1.2258	1.2168	1.2136	1.1524	1.1171	1.1488
	Worst	1.3723	1.2854	1.2582	1.2356	1.1852	1.1554	1.1512
	STD	0.0746	0.0474	0.0314	0.0296	0.0291	0.0245	0.0195
10	Best	1.2346	1.2251	1.2014	1.1987	1.1912	1.1847	1.1654
	Mean	1.3387	1.2866	1.2755	1.2441	1.2241	1.2014	1.1988
	Worst	1.3987	1.3541	1.3065	1.2907	1.2547	1.2465	1.2247
	STD	0.0841	0.0674	0.0544	0.0476	0.0324	0.0321	0.0287
11	Best	1.2014	1.2156	1.1923	1.1904	1.1674	1.1541	1.1423
	Mean	1.3044	1.3209	1.3121	1.2946	1.2213	1.2009	1.1846
	Worst	1.3974	1.3874	1.3564	1.3465	1.2935	1.2756	1.2634
	STD	0.0985	0.0865	0.0848	0.0794	0.0631	0.0612	0.0614
12	Best	1.2341	1.2285	1.1954	1.1695	1.1674	1.1547	1.1498
	Mean	1.3155	1.3066	1.2466	1.1922	1.1899	1.1714	1.1655
	Worst	1.3741	1.3654	1.2784	1.2354	1.2241	1.2014	1.1987
	STD	0.0714	0.0693	0.0420	0.0345	0.0305	0.0246	0.0251
13	Best	1.2045	1.2236	1.1836	1.1582	1.1554	1.1456	1.1421
	Mean	1.3246	1.3068	1.2531	1.1758	1.1621	1.1570	1.1795
	Worst	1.3877	1.3756	1.3455	1.2765	1.2395	1.2353	1.2236
	STD	0.0930	0.0761	0.0712	0.0638	0.0553	0.0544	0.0407
14	Best	1.2289	1.2354	1.2036	1.1751	1.1756	1.1714	1.1654
	Mean	1.3011	1.2765	1.2566	1.2014	1.1967	1.1984	1.1852
	Worst	1.3687	1.3574	1.3168	1.2541	1.2462	1.2387	1.2146
	STD	0.0684	0.0613	0.0564	0.0407	0.0384	0.0345	0.0254
15	Best	1.2056	1.1987	1.1823	1.1643	1.1612	1.1548	1.1423
	Mean	1.2960	1.2486	1.2209	1.1896	1.1869	1.1608	1.1531
	Worst	1.3785	1.3564	1.2964	1.2563	1.2236	1.1952	1.1923
	STD	0.0861	0.0813	0.0585	0.0471	0.0310	0.0217	0.0261
16	Best	1.2454	1.2214	1.2036	1.1874	1.1746	1.1574	1.1473
	Mean	1.3022	1.2658	1.2514	1.2144	1.1945	1.1854	1.1532
	Worst	1.3695	1.3541	1.2874	1.2541	1.2245	1.2019	1.1895
	STD	0.0658	0.0678	0.0421	0.0375	0.0284	0.0228	0.0227
17	Best	1.2065	1.1886	1.1854	1.1822	1.1562	1.1432	1.1054
	Mean	1.2881	1.2397	1.2501	1.2333	1.1725	1.1989	1.1200
	Worst	1.3563	1.3265	1.3074	1.2854	1.2534	1.2254	1.1754
	STD	0.0754	0.0698	0.0616	0.0586	0.0523	0.0431	0.0365
18	Best	1.2146	1.2236	1.1987	1.1741	1.1712	1.1689	1.1612
	Mean	1.2987	1.2977	1.2411	1.2050	1.1897	1.1823	1.1754
	Worst	1.3754	1.3574	1.2741	1.2414	1.2341	1.2241	1.2146
	STD	0.0898	0.0675	0.0394	0.0348	0.0474	0.0274	0.0245
19	Best	1.2063	1.2136	1.1932	1.1632	1.1563	1.1524	1.1423
	Mean	1.2955	1.2801	1.2274	1.1961	1.1983	1.1801	1.1780
	Worst	1.3892	1.3541	1.3014	1.2756	1.2569	1.2365	1.2136
	STD	0.0918	0.0712	0.0643	0.0598	0.0558	0.0430	0.0369
20	Best	1.2121	1.2036	1.2021	1.1754	1.1724	1.1532	1.1222
	Mean	1.2977	1.2870	1.2440	1.2122	1.2193	1.1790	1.1404
	Worst	1.3756	1.3687	1.3214	1.2874	1.2833	1.2333	1.1874
	STD	0.0887	0.0814	0.0641	0.0556	0.0547	0.0414	0.0374

**Table 4:** Performance variation over varying numbers of PCCGWO’s slaves: SLR criterion.

Scenario	Obstacles	Number of Slaves in the PCCGWO Algorithms					
		2	4	6	8	10	12
1	40	1.2488	1.2402	1.1923	1.1852	1.1653	1.1631
2	45	1.2671	1.2612	1.2079	1.1952	1.1680	1.1641
3	50	1.2967	1.2883	1.2119	1.2075	1.1978	1.1956
4	55	1.3181	1.2977	1.2172	1.2135	1.2147	1.2113
5	60	1.3483	1.3187	1.2467	1.2329	1.2245	1.2154
6	65	1.2870	1.2440	1.2122	1.2193	1.1790	1.1404
7	70	1.3714	1.3228	1.2603	1.2457	1.2251	1.2240
8	75	1.4070	1.3504	1.2695	1.2567	1.2274	1.2258
9	80	1.5832	1.3569	1.2716	1.2630	1.2317	1.2279
10	85	1.5929	1.3584	1.2874	1.2801	1.2585	1.2490

**Table 5:** Friedman’s ranking of the algorithms for mean performance: standardized cost criterion.

Scenarios	Algorithms						
	GWO	PCCGWO-2	PCCGWO-4	PCCGWO-6	PCCGWO-8	PCCGWO-10	PCCGWO-12
	Rank	Rank	Rank	Rank	Rank	Rank	Rank
1	5	4	1	6	2	3	7
2	7	6	5	3	2	4	1
3	7	6	5	4	3	2	1
4	7	6	5	4	3	2	1
5	7	3	2	5	4	6	1
6	7	6	5	4	3	2	1
7	7	6	5	4	3	2	1
8	7	6	5	4	3	1	2
9	7	6	5	4	3	1	2
10	7	6	5	4	3	2	1
11	7	6	5	4	3	2	1
12	7	6	5	4	3	2	1
13	7	6	5	2	3	1	4
14	7	6	5	4	2	3	1
15	7	6	5	3	2	4	1
16	7	6	5	4	3	2	1
17	5	4	7	6	2	3	1
18	7	6	5	4	3	1	2
19	7	6	5	4	3	2	1
20	7	6	4	2	5	3	1
Ranks’ sum	136	113	94	79	58	48	32

**Table 6:** Friedman’s ranking of the algorithms for mean performance: SLR criterion.

Scenarios	Algorithms						
	GWO	PCCGWO-2	PCCGWO-4	PCCGWO-6	PCCGWO-8	PCCGWO-10	PCCGWO-12
	Rank	Rank	Rank	Rank	Rank	Rank	Rank
1	6	5	1	2	3	4	7
2	7	6	5	4	3	2	1
3	7	6	5	4	3	2	1
4	7	6	5	4	3	2	1
5	7	3	2	5	4	6	1
6	7	6	5	4	3	2	1
7	7	6	5	4	3	2	1
8	7	6	5	4	3	2	1
9	7	6	5	4	3	1	2
10	7	6	5	4	3	2	1
11	5	7	6	4	3	2	1
12	7	6	5	4	3	2	1
13	7	6	5	3	2	1	4
14	7	6	5	4	2	3	1
15	7	6	5	4	3	2	1
16	7	6	5	4	3	2	1
17	7	5	6	4	2	3	1
18	7	6	5	4	3	2	1
19	7	6	5	3	4	2	1
20	7	6	5	3	4	2	1
Ranks’ sum	137	116	95	76	60	46	30

**Table 7:** Paired comparison of the proposed algorithms: standardized cost criterion.

	PCCGWO-2	PCCGWO-4	PCCGWO-6	PCCGWO-8	PCCGWO-10	PCCGWO-12
**GWO**	23	42	57	78	88	104
**PCCGWO-2**	–	19	34	55	65	81
**PCCGWO-4**	–	–	15	36	46	62
**PCCGWO-6**	–	–	–	21	31	47
**PCCGWO-8**	–	–	–	–	10	26
**PCCGWO-10**	–	–	–	–	–	16

**Table 8:** Paired comparison of the proposed algorithms: SLR criterion.

	PCCGWO-2	PCCGWO-4	PCCGWO-6	PCCGWO-8	PCCGWO-10	PCCGWO-12
**GWO**	21	42	61	77	91	107
**PCCGWO-2**	–	21	40	56	70	86
**PCCGWO-4**	–	–	19	35	49	65
**PCCGWO-6**	–	–	–	16	30	46
**PCCGWO-8**	–	–	–	–	14	30
**PCCGWO-10**	–	–	–	–	–	16

**Table 9:** Computational time measurement of PCCGWO algorithms: CT metric (sec).

Scenario		GWO	PCCGWO-2	PCCGWO-4	PCCGWO-6	PCCGWO-8	PCCGWO-10	PCCGWO-12
1	Best	911.5413	305.5142	120.3264	95.8741	71.2225	63.2145	60.9884
	Mean	919.3462	308.4683	123.6590	97.1411	73.4176	64.7595	61.5689
	Worst	930.2144	312.2636	126.3254	99.2236	75.2214	66.2111	63.1114
	STD	9.3403	3.3781	3.0112	1.5589	2.0226	1.4324	0.5781
2	Best	1321.3214	378.2141	138.2143	109.3254	78.1412	67.7841	64.7412
	Mean	1345.6521	387.2136	141.3154	112.2165	79.6541	69.1252	66.2143
	Worst	1378.5113	398.3214	146.2541	115.0536	81.9654	70.9143	67.9412
	STD	28.7044	10.0721	2.5465	2.5077	1.2188	0.9677	0.7899
3	Best	1543.3652	421.2145	151.8874	124.9412	84.1521	74.6521	68.2541
	Mean	1556.2145	425.2314	154.2142	126.2143	85.2541	75.2845	69.3251
	Worst	1565.4133	428.2541	156.9984	128.5471	87.5241	76.2214	70.5241
	STD	11.0471	3.5347	2.5541	1.5305	1.5241	0.7112	0.7014
4	Best	1675.3254	474.6251	173.5412	135.2146	90.2143	78.8965	69.1745
	Mean	1689.2135	479.2143	175.9852	137.1456	91.3264	79.6541	70.2143
	Worst	1700.3652	483.2145	177.6399	139.8854	92.6541	80.6231	71.2541
	STD	12.5473	3.7941	2.0674	2.3489	1.2287	0.8674	0.7114
5	Best	1819.3214	515.2365	188.3146	147.2514	95.2256	84.2145	78.2254
	Mean	1826.7733	518.0455	191.6003	149.0560	97.4111	86.7754	79.0130
	Worst	1830.6214	522.3651	193.2146	151.2223	98.5874	88.2146	80.3365
	STD	17.2485	3.5874	2.7789	1.90321	1.7223	2.0261	1.0558
6	Best	2066.3265	576.3652	196.3254	160.3214	101.3325	91.6541	82.5413
	Mean	2076.3265	579.8523	198.2146	162.3264	102.3214	92.3641	83.6524
	Worst	2089.3254	583.6521	201.3254	164.3265	103.9987	93.1234	84.8741
	STD	11.5002	3.6474	2.5247	2.0074	1.3414	0.7341	1.1204
7	Best	2174.2541	651.2146	212.3265	173.2651	106.5241	97.5562	85.5527
	Mean	2183.1258	654.3214	215.2463	175.8543	108.2541	98.5141	86.5412
	Worst	2190.3265	659.5446	217.9985	178.3325	110.3254	99.8574	87.2141
	STD	8.0014	4.2005	2.8874	2.5374	1.9044	1.1511	0.8374
8	Best	2355.9852	698.2541	226.3264	187.3254	110.3652	102.3254	89.6542
	Mean	2365.2654	702.3614	228.9874	188.6413	111.2365	103.6521	90.3652
	Worst	2378.7141	706.5234	230.3214	190.3265	112.6897	104.9852	91.2541
	STD	11.4632	4.1332	2.0312	1.0112	1.1798	1.3205	0.8074
9	Best	2535.6231	758.2541	238.2541	197.2314	114.2289	109.8741	95.2148
	Mean	2548.1259	762.6293	240.2468	199.4514	116.3322	110.8850	96.6493
	Worst	2555.3251	765.2365	243.2561	201.2315	118.5698	111.6548	97.8854
	STD	9.9021	3.8741	2.4412	2.0053	2.1002	0.8741	1.3365
10	Best	2752.3251	807.3254	259.8741	207.2146	122.6652	112.8745	103.6654
	Mean	2765.3254	812.3254	262.3241	209.3652	123.6521	113.2146	104.2143
	Worst	2774.6324	816.3251	264.6521	210.3265	124.8974	114.1236	105.1235
	STD	11.2987	4.5114	2.3874	1.5998	1.1173	0.6474	0.7314
11	Best	2884.3651	838.6251	279.8412	215.2314	126.2541	114.3241	109.052
	Mean	2898.9306	848.9878	288.0397	217.2632	128.7884	115.5282	110.0093
	Worst	2895.3214	854.2341	292.3641	220.3214	130.2654	116.3541	111.3264
	STD	7.5032	7.7242	6.7651	2.8254	2.0125	1.0144	0.8854
12	Best	3009.2314	1054.3241	305.3254	226.3254	135.4232	123.6524	117.7413
	Mean	3015.6472	1057.2657	308.6874	227.8542	136.2143	124.6521	118.3214
	Worst	3024.2134	1061.3241	310.2314	229.6541	137.6541	125.9874	119.6243
	STD	7.3254	3.5174	2.1871	1.6677	1.1374	1.1701	0.9601
13	Best	3276.2156	1135.3621	317.2156	231.2541	140.3256	129.3254	121.5563
	Mean	3285.9941	1145.9659	321.2547	235.7442	142.3955	131.2394	123.2458
	Worst	3295.2596	1151.3214	327.3215	237.8213	145.3652	133.2231	124.3326
	STD	9.2143	8.1456	5.0793	3.4687	2.0354	1.9231	1.3231
14	Best	3365.3210	1204.6652	336.5412	243.6541	147.1123	131.4152	126.8745
	Mean	3371.3652	1208.3652	338.5413	245.6521	148.3214	132.2145	127.3264
	Worst	3381.3250	1213.3254	340.6523	246.9985	149.8993	133.6541	128.5541
	STD	8.0143	3.3001	2.0998	1.6822	1.3941	1.1319	0.8602
15	Best	3425.2231	1251.2134	349.3215	250.3214	154.3321	133.6998	131.3264
	Mean	3432.5063	1264.4021	352.8324	254.0122	155.2483	134.4023	132.1470
	Worst	3440.5231	1269.5874	356.2143	257.2145	157.3254	135.6252	133.6524
	STD	7.1123	9.8774	3.4887	3.8857	1.5228	1.1712	0.9712
16	Best	3791.2513	1144.3265	374.8871	257.3241	161.2445	137.8741	136.8874
	Mean	3798.3254	1146.6541	376.9852	259.3652	162.3254	138.5241	137.3264
	Worst	3808.2365	1151.8521	378.9236	261.3265	163.8745	139.6412	138.9841
	STD	8.1326	2.7102	2.0100	2.0088	1.3204	0.8901	1.1036
17	Best	3959.3214	1325.2141	387.2145	264.6325	168.2541	144.9985	141.3336
	Mean	3964.5898	1335.9107	392.1166	266.0347	169.4657	145.4018	142.3379
	Worst	3975.3256	1341.2365	396.3219	268.3214	171.3265	147.3261	142.9745
	STD	8.1001	6.7789	4.4123	3.8514	1.5142	1.2487	0.8214
18	Best	4176.6541	1394.3254	405.3254	296.5241	173.8974	154.8764	147.8541
	Mean	4189.6312	1399.5413	407.3267	298.7413	174.6652	155.8032	148.3621
	Worst	4196.3214	1405.3214	410.3652	301.2354	175.8743	156.4123	149.1365
	STD	9.4567	5.5087	2.5374	2.3774	0.9974	0.7778	0.7727
19	Best	4312.3261	1456.3257	412.3214	324.2314	177.3265	163.2523	154.5413
	Mean	4322.3891	1461.5171	416.5431	326.2189	178.7762	164.5179	155.6984
	Worst	4331.3251	1476.3652	422.3256	329.3254	179.3254	165.3288	156.3654
	STD	9.0351	6.9974	4.2223	3.5541	1.0389	1.0141	0.9190
20	Best	4349.5412	1469.3254	431.2213	305.2314	186.8541	172.2235	167.8945
	Mean	4358.7562	1473.0308	434.7432	307.9164	188.6586	173.1247	168.9587
	Worst	4365.3254	1476.2541	436.2214	309.6685	189.9845	174.6547	169.6852
	STD	7.0370	3.4226	2.5447	2.2668	1.5747	1.2874	0.9114

**Table 10:** Path length under varying iterations and population sizes of the problem (4).

Max Iter	Pop	Path Length (km)						
		GWO	PCCGWO-2	PCCGWO-4	PCCGWO-6	PCCGWO-8	PCCGWO-10	PCCGWO-12
1500	1200	104.8208	103.9609	100.4901	97.9196	98.4952	95.2382	92.1185
	1600	104.8161	103.9124	100.4175	97.6541	97.8856	95.1867	91.9874
	2000	104.7852	102.4171	100.2145	97.1423	96.8451	95.1022	91.4213
2000	1200	104.7611	105.5241	100.6524	96.5240	96.6477	95.0536	90.8741
	1600	104.7452	104.9640	100.5241	96.1234	95.5431	94.9741	90.1234
	2000	104.7366	104.5231	100.4123	95.7441	94.3654	94.7541	89.9748
2500	1200	104.7014	108.9521	100.6974	95.1243	93.4271	94.5747	89.4574
	1600	104.6974	108.6241	100.6142	94.6541	92.7841	94.4123	88.9874
	2000	104,6841	108.5346	100.5978	93.3103	91.4484	94.2098	88.1024

**Table 11:** Computational time under varying iterations and population sizes of the problem (4).

Max Iter	Pop	Computational Time (sec)						
		GWO	PCCGWO-2	PCCGWO-4	PCCGWO-6	PCCGWO-8	PCCGWO-10	PCCGWO-12
1500	1200	4358.7562	1473.0308	434.7432	307.9164	188.6586	173.1247	168.9587
	1600	5874.3251	1712.0402	547.3584	370.5632	220.2547	204.6525	189.6521
	2000	6587.3256	1998.6414	638.7512	401.5741	279.6514	256.3241	220.4512
2000	1200	7854.5567	2345.2411	786.8225	489.5127	301.4276	291.2354	260.5411
	1600	8752.3389	2687.5418	865.1140	578.1143	356.8123	324.3521	298.6278
	2000	9687.5241	2871.8892	974.6823	647.3328	387.4412	365.3248	335.5741
2500	1200	10475.531	3564.5241	1000.7412	698.3241	412.3641	398.6541	367.8749
	1600	11541.317	4100.5241	1107.5241	745.6231	487.3364	465.3654	435.9871
	2000	12081.541	4340.5618	1262.6289	873.7593	537.1352	483.0177	445.5718

**Table 12:** Performance comparison of the PCCGWO-12 algorithm with recent metaheuristics.

Algorithms										
	WCA		SSA		CSA		MVO		PCCGWO-12	
	SLR	CT	SLR	CT	SLR	CT	SLR	CT	SLR	CT
Best	1.2865	7636.214	1.3126	13758.21	1.4156	3854.654	3.1456	3974.216	1.1222	167.8945
Mean	1.3210	7788.391	1.4470	13859.58	1.5525	3998.179	3.4373	4260.839	1.1404	168.9587
Worst	1.4563	7892.321	1.5569	14014.36	1.7412	4063.541	3.7652	4465.321	1.1874	169.6852
STD	0.0982	112.7417	0.1389	119.0197	0.1741	101.8591	0.3198	204.6947	0.0374	0.9114

## Data Availability

No new data were created or analyzed in this study.
